# Vascular endothelial growth factor C promotes breast cancer progression via a novel antioxidant mechanism that involves regulation of superoxide dismutase 3

**DOI:** 10.1186/s13058-014-0462-2

**Published:** 2014-10-30

**Authors:** Chu-An Wang, J Chuck Harrell, Ritsuko Iwanaga, Paul Jedlicka, Heide L Ford

**Affiliations:** 1 Department of Pharmacology, University of Colorado School of Medicine, Anschutz Medical Campus, 12800 E.19th Ave, Aurora, 80045 CO USA; 2 Lineberger Comprehensive Cancer Center, University of North Carolina at Chapel Hill, 101 Manning Drive, Chapel Hill, 27599 NC USA; 3 Department of Obstetrics and Gynecology, University of Colorado School of Medicine, Anschutz Medical Campus, 12800 E.19th Ave, Aurora, 80045 CO USA; 4 Department of Pathology, University of Colorado School of Medicine, Anschutz Medical Campus, 12800 E.19th Ave, Aurora, 80045 CO USA; 5 Present Address: Institute of Basic Medical Sciences, College of Medicine, National Cheng Kung University, No.1, University Road, Tainan City, 701 Taiwan

## Abstract

**Introduction:**

Triple-negative breast cancers, particularly the claudin-low subtype, are highly aggressive and exhibit increased tumor-initiating cell (TIC) characteristics. In this study, we demonstrate that vascular endothelial growth factor C (VEGF-C) is highly expressed in the claudin-low breast cancer subtype and also that it mediates tumor progression, not only through its role in lymphangiogenesis but also through regulating TIC characteristics and the response to reactive oxygen species (ROS).

**Methods:**

*VEGF C* expression was examined in breast cancer subtypes, and a *VEGF C* expression signature was derived. *VEGF C* expression and/or its associated signature was correlated with TIC and chemoresistance signatures. *In vitro* and *in vivo* assays were performed to determine whether VEGF-C expression alters TIC characteristics and the response of breast cancer cells to chemotherapy and oxidative stress. Array analysis was used to identify a downstream effector of VEGF-C, superoxide dismutase 3 (Sod3), which was tested for its involvement in VEGF-C-mediated resistance to oxidative stress and enhancement of *in vivo* metastasis. The VEGF-C-associated receptor neuropilin 2 (Nrp2) was knocked down to determine whether it is required for the observed effects of VEGF-C. Expression of *VEGF C* and *Sod3* was assessed in human breast cancers.

**Results:**

*VEGF C* is highly expressed in claudin-low breast cancers, and *VEGF C* and the *VEGF C* signature are associated with TIC-related gene signatures. VEGF-C-knockdown in mammary carcinoma cells decreases TIC properties *in vitro* and *in vivo*, sensitizing cells to oxidative stress and chemotherapy. We identified Sod3 as a target of VEGF-C in breast cancer cells by demonstrating that it is required for VEGF-C-mediated cell survival in response to oxidative stress and for VEGF-C-mediated metastasis. We demonstrate that Nrp2 is the VEGF-C-associated receptor that mediates alterations in Sod3 expression and the response of tumor cells to oxidative stress. We show that *VEGF C* and *Sod3* are positively associated in human breast cancer.

**Conclusions:**

We describe a novel mechanism by which VEGF-C contributes to metastasis via its ability to enhance TIC-associated characteristics, particularly the response to ROS. We identified Sod3 as a critical mediator of VEGF-C-induced metastasis, and we provide evidence that the VEGF-C-Sod3 axis plays a role in human breast cancers.

**Electronic supplementary material:**

The online version of this article (doi:10.1186/s13058-014-0462-2) contains supplementary material, which is available to authorized users.

## Introduction

Reactive oxygen species (ROS) can be generated endogenously or from exogenous sources. In normal cells, ROS has important biological functions, such as in the elimination of pathogens. However, excessive levels of ROS can cause damage to cells, in some cases leading to increased mutation rates. Conflicting roles, both stimulatory and inhibitory, have been reported for oxidative stress in cancer progression. Cancer cells have been shown to have increased levels of ROS, and sublethal levels of ROS within these cells can promote proliferation and genomic instability [1],[2]. However, the increased levels of ROS can also make cancer cells more sensitive to ROS-inducing agents such as chemotherapeutic drugs [3]. Thus, to survive under high-stress conditions, a small population of cancer cells which express markers of tumor-initiating cells (TICs) evolve a defense system against ROS [4],[5]. These data suggest that the TIC population is protected against exogenous stress and that the ability to withstand ROS may in part account for tumor recurrence. Thus, understanding the mechanism by which tumor cells acquire antioxidative capabilities, as well as identifying means to block pathways involved in antioxidation, may lead to the discovery of novel therapies that can be used in conjunction with chemotherapy or irradiation.

Vascular endothelial growth factor C (VEGF-C) is the major embryonic lymphangiogenic factor; it is required for the initial migration and sprouting of committed endothelial cells [6],[7]. Overexpression of VEGF-C is observed in numerous cancers [8], and its expression is associated with high lymphatic vessel density and poor survival [9]. A significant amount of evidence supports an active role for VEGF-C in cancers by its induction of tumor-associated lymphangiogenesis, which facilitates tumor cell dissemination to lymph nodes and metastasis to distant organs [10]-[12]. Interestingly, growing evidence suggests that VEGF-C can also contribute to tumor progression in a tumor cell autonomous manner.

The effects of VEGF-C on cancer cells (as opposed to endothelial cells) include its ability to increase tumor cell proliferation, migration and invasion [13]-[17]. Cancer cells in several tumor types are known to express one or more of the VEGF-C-responsive receptors on the cell surface, thereby providing a means for the growth factor to signal directly to the cancer cells. In addition to its effects on solid tumors, VEGF-C can promote the proliferation and survival of leukemia cells after chemotherapy [18]. More recently, inhibition of VEGF-C was shown to decrease mesenchymal markers and increase epithelial markers, as well as to reduce the side population (a marker of cancer stem cells) in lung cancer [19]. Together, these studies suggest that VEGF-C not only induces tumor-associated lymphangiogenesis but also might promote tumor progression through multiple mechanisms that either directly or indirectly affect the cancer cell. Importantly, these studies indicate that VEGF-C may be a potent drug target because of its ability to affect multiple aspects of tumor progression, which encompass both the tumor cells and cells in the tumor microenvironment.

In this study, we demonstrate that VEGF-C is important in maintaining breast TIC populations and that it contributes to the ability of tumor cells to survive under oxidative stress and in response to chemotherapeutic insults. We further identify superoxide dismutase 3 (Sod3), an antioxidant enzyme, as a downstream effector of VEGF-C and show that it is required for the antioxidative function of VEGF-C *in vitro*, as well as for the ability of VEGF-C to mediate tumor growth and metastasis *in vivo*. Our results allow us to define a novel antioxidant function for VEGF-C in breast cancer and elucidate a novel mechanism by which VEGF-C regulates the response to ROS.

## Methods

### Cell lines

To generate VEGF-C-knockdown (KD) or neuropilin 2 (Nrp2) KD in murine 66 cl4 and/or human MDA-MB-231 cells, short-hairpin RNAs (shRNAs) against *VEGFC* or *Nrp2* were purchased (Open Biosystems/GE Dharmacon, Lafayette, CO, USA) and delivered retrovirally according to the manufacturer's protocol. Scramble shRNA obtained from Addgene (Cambridge, MA, USA) or from Open Biosystems/GE Dharmacon were used as controls. Cells containing the KD constructs were selected using puromycin, and the two KD clones that most efficiently reduced VEGF-C/Nrp2 levels were selected for subsequent studies. Sod3 cDNA (CMV-Sport1; Open Biosystems/GE Dharmacon) was cloned into a pCDH lentivector (SBI System Biosciences, Mountain View, CA, USA) and transfected into 66 cl4 VEGF-C KD cells. Stable clones with Sod3 restoration were selected via green fluorescent protein sorting. The pCDH lentivector was also introduced into the 66 cl4 scramble and VEGF-C KD cells to serve as a control. The mouse mammary carcinoma 66 cl4 cell line was kindly provided by Dr. Fred R. Miller, and the MDA-MB-231 cell line was fingerprinted by the University of Colorado Cancer Center DNA sequencing center to ensure that it matched the authentic MDA-MB-231 cell line (November 2011; American Type Culture Collection, Manassas, VA, USA).

### *In vitro*cell-based assays

Annexin V apoptosis assays (BD Biosciences, San Jose, CA, USA) and ALDEFLUOR assays (STEMCELL Technologies, Vancouver, BC, Canada) were performed according to the manufacturers' protocols. In cell viability assays (Molecular Probes/Life Technologies, Eugene, OR, USA), 66 cl4 scramble and VEGF-C KD cells were plated at 10^6^ cells per 10-cm dish, and the next day cells were treated with H_2_O_2_ (1 mM) or H_2_O (control) in serum-free medium for 24 hours. Attached cells and cells in the medium were collected and stained using ethidium homodimer 1 dye for dead cells and calcein AM dye for live cells. The percentage of live and dead cells within the populations was analyzed by flow cytometry. In chemosensitivity assays, cells were plated at 25,000 cells per well (5 replicate wells) in 96-well plates. The following day, cells were treated with varying concentrations of etoposide (Sigma-Aldrich, St Louis, MO, USA) or doxorubicin (Sigma-Aldrich) in serum-free media. After 24 hours, cell viability was assessed using the CellTiter-Glo assay (Promega, Madison, WI, USA). For clonogenic assays, 1,000 cells per well were plated in triplicate in 6-well plates. The following day, cells were treated with doxorubicin or phenethyl isothiocyanate (PEITC) in serum-free media. After 24 hours, cells were washed and fresh medium (with complete serum) was added. After 2 weeks, colonies were stained with crystal violet and counted visually.

### Real-time PCR

All analyses were performed using the CFX96 real-time PCR detection system (Bio-Rad Laboratories, Hercules, CA, USA). TaqMan primers used against mouse and human *VEGFC* were purchased from Applied Biosystems (Foster City, CA, USA), and TaqMan primer against mouse *Nrp2* was purchased from Integrated DNA Technologies (Coralville, IA, USA). The SYBR Green assay primers for *Sod3* were as follows: murine *Sod3*, forward: GCTCTTGGGAGAGCCTGAC, and reverse: GGTCAAGCCTGTCTGCTAGG; human *SOD3*, forward: CAGGAGAGAAAGCTCTCTTGGA, and reverse: GAGCAGGCAGGAACACAGTAG. Relative expression was normalized to the expression of cyclophilin B in the cells. A mouse oxidative quantitative PCR array was purchased (QIAGEN, Valencia, CA, USA) and analyzed according to manufacturer's instructions.

### Western blot analysis

Whole-cell lysates were collected with radioimmunoprecipitation assay buffer, and medium from cells was concentrated using a centrifugal filter unit (EMD Millipore, Billerica, MA, USA). VEGF-C and Sod3 antibodies (sc-25783 and sc-67089, respectively) were obtained from Santa Cruz Biotechnology (Santa Cruz, CA, USA). Antibody against human SOD3 was obtained from Novus Biologicals (NBP1-22417; Littleton, CO, USA). Antibody against NRP2 was obtained from Cell Signaling Technology (D39A5; Danvers, MA, USA).

### *In vivo*experiments

Six- to eight-week-old female BALB/c mice were purchased from the National Cancer Institute (Rockville, MD, USA). For the orthotopic experiment, 1 × 10^6^ cells in 100 μl of Dulbecco's modified Eagle's medium were injected into the fourth mammary fat pad. Tumor growth was measured weekly using calipers, and *in vivo* imaging was performed weekly as previously described [20]. Animal protocols performed in this study were approved by the Institutional Animal Care and Use Committee at the University of Colorado Anschutz Medical Campus.

### Statistical analysis

Statistical analyses were performed by two-tailed *t*-test for comparing two groups. One-way analysis of variance (ANOVA) with Tukey's posttests were performed for comparing more than three groups. Two-way ANOVA with Bonferroni posttests were performed for tumor growth and cell viability analyses. χ^2^ and Fisher's exact tests were performed for tumor formation analyses noted in the figure legends. GraphPad Prism 5 software (GraphPad Software, La Jolla, CA, USA) was used to perform the analyses. Error bars represent the standard error of the mean of three independent experiments. Asterisks denote significant differences from control groups (*P < 0.05; **P < 0.01; ***P < 0.001).

## Results

### *VEGF C*expression is high in the claudin-low subtype of breast cancer and correlates with gene expression signatures associated with poor clinical outcome

**Figure 1:**
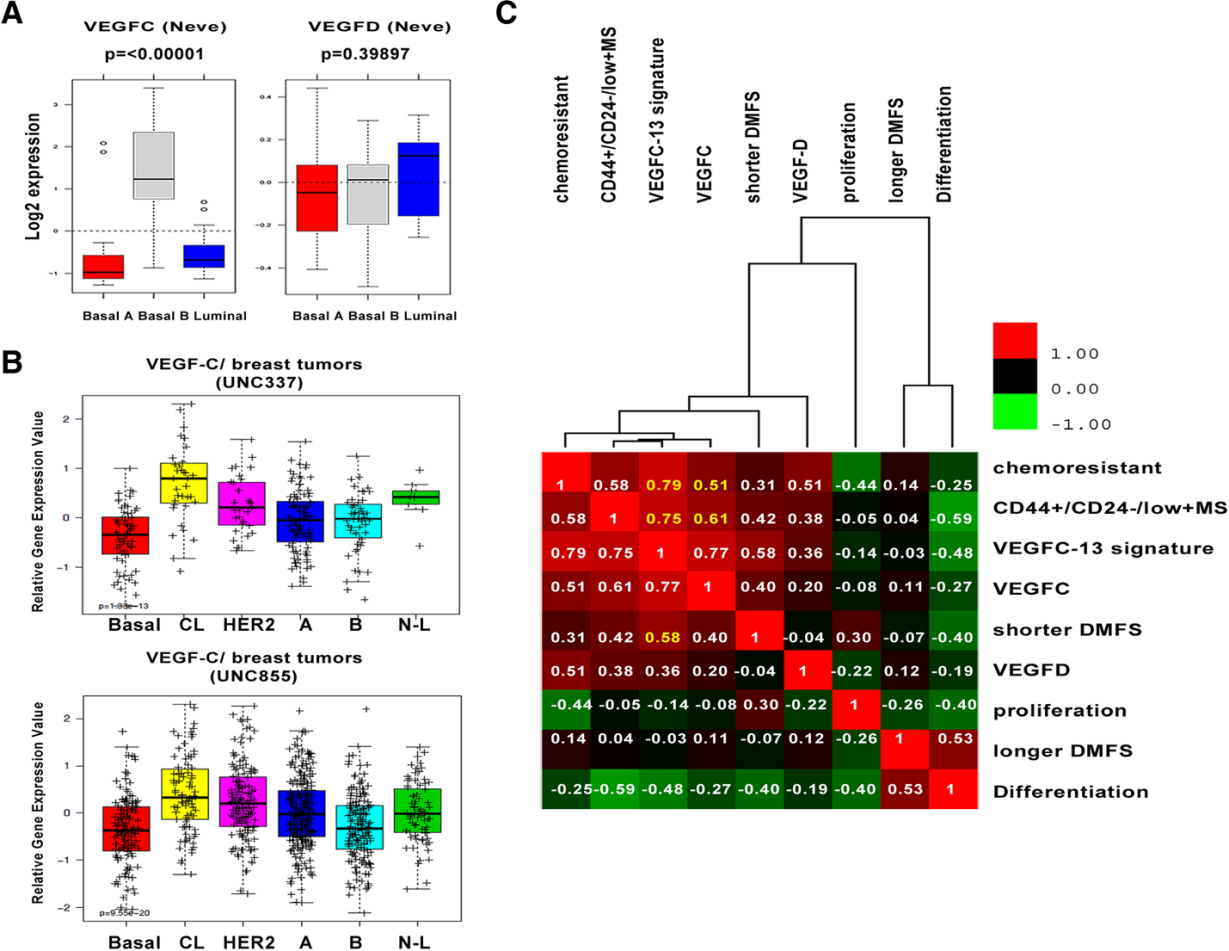
**Vascular endothelial growth factor C mRNA expression in human breast cancer cell lines and breast tumors. (A)** Box plots of *VEGFC* and *VEGFD* gene expression across 51 previously reported breast cancer cell lines grouped into basal A, basal B and luminal subgroups (left). Vascular endothelial growth factor C (*VEGF C*) expression in human breast cancer cell lines was assessed using GOBO [21]. **(B)** Gene expression data from 337 and 855 human breast tumors (UNC337 and UNC855). Log_2_-transformed and median centered gene expression values for *VEGF C* across the intrinsic subtypes, including basal, claudin-low (CL), human epidermal growth factor receptor 2 (HER2)-enriched (HER2), luminal A (A), luminal B (B) and normal-like (N-L) breast tumors. **(C)** Pearson's correlation values for each gene signature compared to each other for 337 human breast tumors (UNC337 data set). Color scale bars represent Pearson's correlations: *r* =1 (red), *r* = 0 (black) and *r* = 1 (green). DMFS, distant metastasis free survival. MS, mammosphere.

We previously found that VEGF-C KD in 66 cl4 mouse mammary carcinoma cells not only influences distant metastasis when these cells are orthotopically injected into immune competent mice, but also, surprisingly, decreases the primary tumor size [20]. These data suggested to us that VEGF-C may play a role outside its normally described role in lymphangiogenesis, because lymphangiogenesis is believed to serve as a route for metastasis but has not been shown to correlate with tumor growth. Although *VEGF C* expression has been widely demonstrated to correlate with lymph node status and poor prognosis, its expression in specific subtypes of breast cancer has not been extensively examined. Examination of *VEGF C* expression in the Neve *et al*. cell line microarray data set [21],[22] revealed that, in contrast to its highly related family member *VEGFD*, *VEGF C* is expressed predominantly in basal B breast cancer cell lines (Figure 1A). Basal B breast cancer cells have been shown to display mesenchymal/stem cell–like characteristics and to be more invasive *in vitro* [22],[23], and they are considered to be most closely related to the claudin-low intrinsic subtype of human breast cancer [24]. The claudin-low subtype of breast cancer is similarly characterized by stem cell and epithelial-to-mesenchymal transition (EMT)-like features and, most importantly, correlates with a poor prognosis [24],[25]. Analysis of two UNC MicroArray Database [26] data sets, which comprise 337 and 855 breast tumor samples, respectively [24],[27], demonstrates that enriched VEGF-C mRNA expression occurs in the claudin-low subtype of breast tumors compared to all other subtypes (Figure 1B). The majority of claudin-low tumors (61% to 71%) fall into the clinical classification of triple-negative breast cancers [24], which lack expression of the estrogen receptor (ER), the progesterone receptor (PR) and the human epidermal growth factor receptor 2 (HER2), and are aggressive cancers that confer poor clinical outcomes. Because triple-negative breast cancers do not have higher levels of lymphangiogenesis than other breast cancer subtypes [28],[29], these data support the hypothesis that expression of VEGF-C may mediate aggressive phenotypes outside its effect on lymphatic vessel growth.

On the basis of the observation that *VEGF C* is predominately expressed in the claudin-low subtype of breast cancer, which is known to have TIC features, we next sought to determine whether *VEGF C* expression correlates with a TIC or cancer stem cell signature previously created using CD44+/CD24− expression [30],[31]. Indeed, *VEGF C* expression was found to correlate with the CD44+/CD24-low + mammosphere (MS) signature (Pearson's *r* = 0.61) (Figure 1C) [31] and, importantly, also with gene sets that are upregulated in breast tumors after chemotherapy (Pearson's *r* = 0.51) (Figure 1C) [32].

Although the function of VEGF-C has been studied extensively, the broader gene expression programs that mediate its function have not been elucidated fully. To identify the genes that most strongly parallel *VEGF C* expression, Pearson's correlation values were identified for *VEGF C* and each gene that was present in a panel of human breast cancer cell lines [33] and tumors [34]. In total, 13 genes had positive Pearson's correlation values >0.5, indicating that these genes exhibit similar gene expression profiles (*VEGF C*-13 signature) (Additional file 1: Figures S1A and S1B). We found that this signature, like *VEGF C* alone, was expressed predominantly in the claudin-low subtype of breast cancer in two different data sets (Additional file 1: Figures S1C and S1D). Interestingly, our newly derived *VEGF C*-13 signature is more strongly correlated with CD44+/CD24 low + MS and chemoresistance signatures (*r* = 0.75 and *r* = 0.79, respectively) than *VEGF C* alone, indicating that broader gene expression profiles are more efficient than single genes in capturing biological programs. We further found that the *VEGF C*-13 signature also correlates with gene sets associated with shortened metastasis-free survival (*r* = 0.58) [30] (Figure 1C). Thus, our data suggest that *VEGF C* and its related signature are strong indicators of aggressive subtypes of breast cancer and of poor clinical outcomes. They further suggest that VEGF-C may drive not only a lymphangiogenic program but also a program associated with TICs.

### *VEGF C*expression is enriched in TIC and is necessary to maintain ALDH-positive TIC population

**Figure 2:**
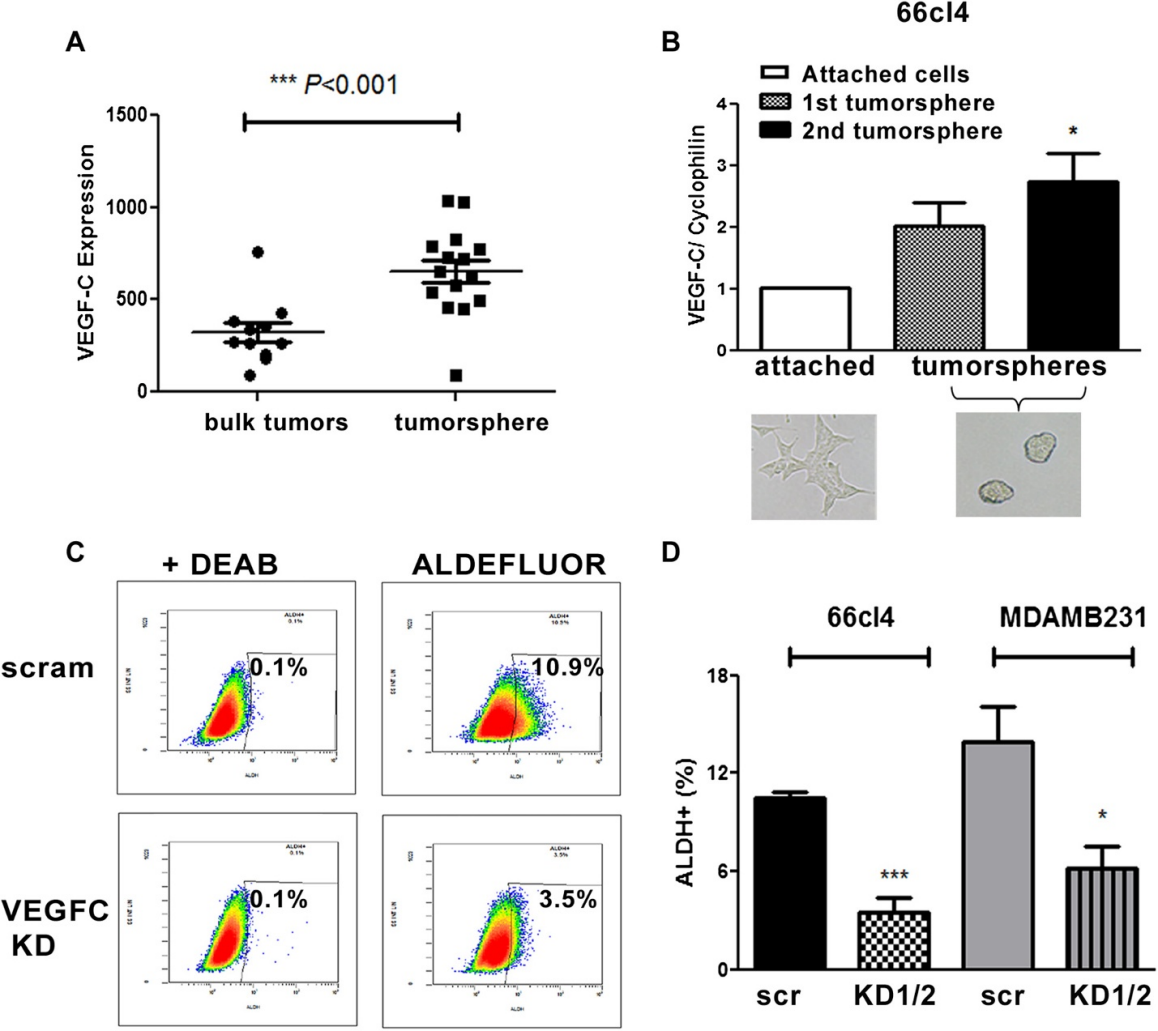
**Vascular endothelial growth factor C-knockdown decreases the aldehyde dehydrogenase-positive tumor-initiating cell population. (A)** Expression of vascular endothelial growth factor C (VEGF-C) mRNA in bulk tumors or tumorspheres formed from cells of primary breast tumors. Gene expression levels were obtained from public microarray data sets [31]. **(B)** Expression of VEGF-C in attached parental cells or tumorspheres formed by growing 66 cl4 cells in serum-free suspension conditions. Pictures of attached cells and tumorspheres are shown. **(C)** Representative flow cytometric data in an ALDEFLUOR assay. Diethylaminobenzaldehyde (DEAB) is a specific inhibitor of aldehyde dehydrogenase (ALDH), which was used as a gating control. **(D)** Quantification of the ALDH-positive population in scramble control (scr) and VEGF-C KD lines from 66 cl4 and MDA-MB-231 cells. Data were combined from three independent experiments using one scramble KD and two VEGF-C KD (KD 1 and 2) cell lines for both 66 cl4 and MDA-MB-231 cells.

In breast cancer, TICs are thought to make up a small proportion of the cells within a tumor; however, importantly, they have been shown to have the ability to self-renew, to establish new tumors and to contribute to tumor heterogeneity. Because we observed that *VEGF C* expression correlates with a breast TIC-related signature, we reasoned that VEGF-C may play a functional role in promoting breast cancer TICs. Analysis of microarray data sets obtained from clinical patient breast tumors [31] demonstrated that VEGF-C mRNA is enriched in tumorspheres formed from cells of primary breast tumors compared to that in the bulk tumors (Figure 2A). Similarly, we detected enrichment in the expression of *VEGF C* in tumorspheres formed from multiple breast cancer cell lines compared to expression in their adherent counterparts (Figure 2B and Additional file 2: Figure S2). Together, these data suggest that expression of *VEGF C* may contribute to TIC characteristics in breast cancer cells. To assess whether VEGF-C influences TIC populations, we performed fluorescence-activated cell sorting analysis for the aldehyde dehydrogenase (ALDH)-positive population, whose activity is used as a surrogate marker for TICs [35], in breast cancer cells with or without VEGF-C KD. Figure 2C shows a significant decrease in ALDH-positive cells in 66 cl4 VEGF-C KD cells compared to the control KD cells (Figure 2D) (Additional file 3: Figure S3). We performed VEGF-C KD in an additional cell line, MDA-MB-231, a basal B human breast cancer cell line that highly expresses VEGF-C receptors (Additional file 4: Figure S4) [14], and, similarly, we observed that the ALDH-positive population was significantly decreased (Figure 2D). These results suggest that VEGF-C expression may be important in maintaining the breast TIC population.

### VEGF-C knockdown leads to decreased tumor initiation and growth *in vivo*, but does not affect proliferation or apoptosis

**Figure 3:**
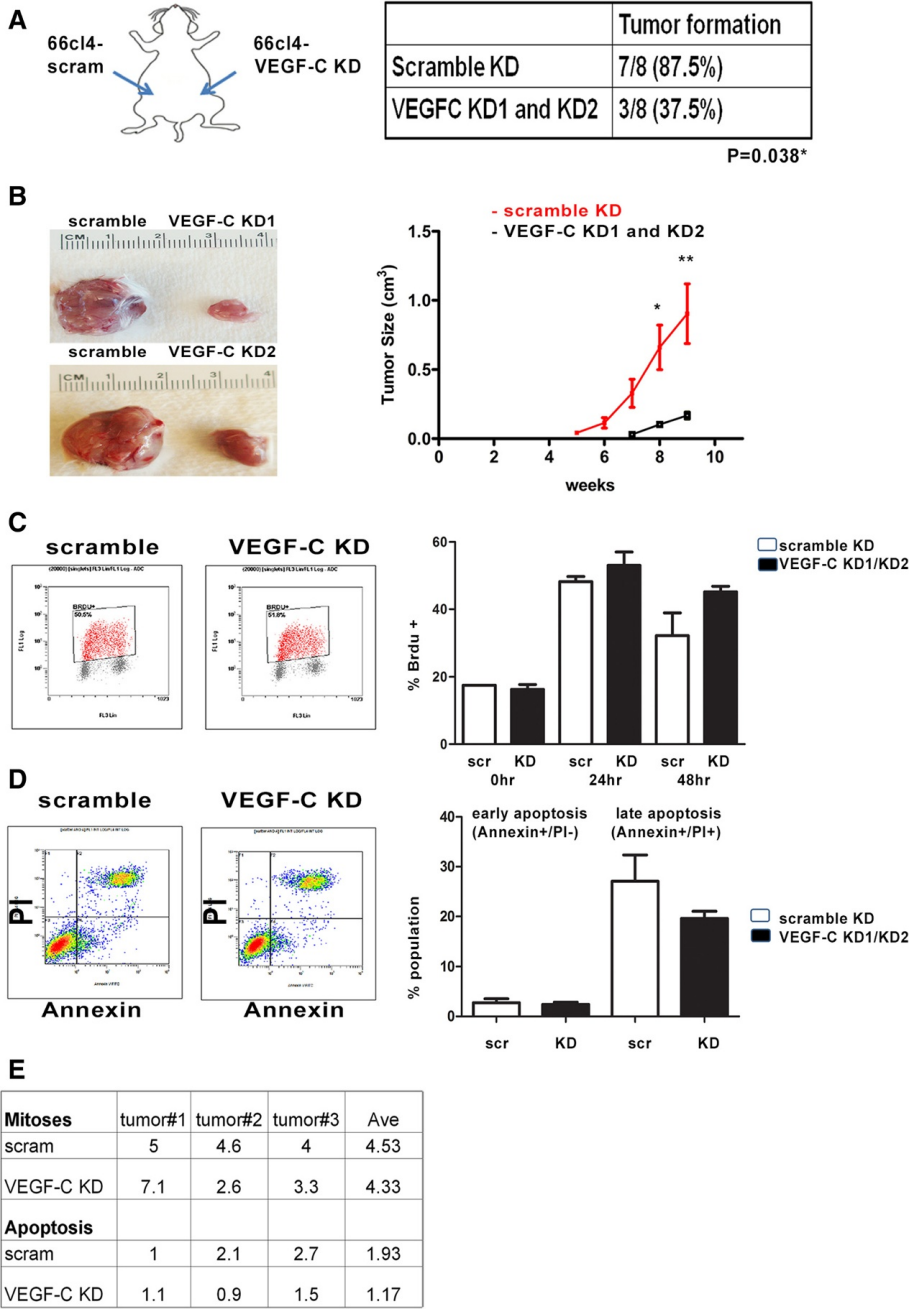
**Vascular endothelial growth factor C-knockdown in 66 cl4 mouse mammary carcinoma cells decreases tumor growth and tumor formation** ***in vivo*** **. (A)** Tumor formation efficiency of ten cells from 66 cl4-scram and 66 cl4-VEGF-C KD1/2 cells injected into the left and right mammary fat pads of female BALB/c mice. The experiment was ended at week 9 after injection because of the large primary tumor sizes in the control group. scr, Scramble; VEGF-C KD, Vascular endothelial growth factor C knockdown. **(B)** Representative picture of 66 cl4-scram and 66 cl4-VEGF-C KD1 and KD2 tumors derived from the left and right mammary fat pads of the same animal (left). Tumor growth in the mice was measured using calipers and calculated using the formula *V* = 1/2(W)(W)(L) (right). *P < 0.05; **P < 0.01. **(C)** Representative flow data show results of a bromodeoxyuridine (BrdU) assay performed on 66 cl4-scram cells, as well as on VEGF-C KD1 and KD2 cells, combined as KD1/2 (left). BrdU-positive populations from 66 cl4-scram and VEGF-C KD1/2 cells at 0, 24 and 48 hours after release from serum starvation are shown. Data were quantified from two independent experiments with duplicates in the control and two different short-hairpin RNA cell lines. **(D)** Representative flow data from a fluorescein isothiocyanate-annexin V apoptosis assay (left). Quantification of the apoptosis assay is shown from two independent experiments with duplicates for detection of the early and late apoptotic populations in the 66 cl4-scram and VEGF-C KD1 and KD2 cells (data combined as KD1/KD2). PI, Propidium Iodide. **(E)** Quantification of mitotic and apoptotic cells is shown from 66 cl4-scram and 66 cl4-VEGF-C KD tumors. Mitotic and apoptotic cells were counted in ten high-power fields per hematoxylin and eosin-stained section. Three control and three VEGF-C KD tumors were counted and analyzed in total. Ave, Average.

We next examined the effect of VEGF-C on *in vivo* breast tumor initiation. Using our previously established 66 cl4-scramble control (scram) and 66 cl4-VEGF-C KD cells (Additional file 4: Figure S4) [20], ten cells of each cell line were injected into the left and right abdominal mammary fat pads of immunocompetent BALB/c mice (as shown in Figure 3A). At week 9, we observed that mammary fat pads injected with 66 cl4-scram cells formed tumors with 87.5% efficiency (7/8), whereas VEGF-C KD cells formed tumors with only 37.5% efficiency (3/8) (*P = 0.038, χ^2^ test) (Figure 3A). In addition, tumors that arose in the context of 66c14-VEGF-C KD were significantly smaller and grew less compared to 66 cl4-scram tumors over time (Figure 3B), which is similar to what we had previously observed when higher cell numbers were injected [20]. Despite the dramatic difference in size between 66 cl4-scram and 66 cl4-VEGF-C KD tumors, we did not observe significant alterations in proliferation or apoptosis between these cells *in vitro* or *in vivo* (Figures 3C to 3E). Together, our findings suggest that VEGF-C affects tumor initiation and growth through increasing the TIC population in breast cancer.

### VEGF-C protects breast cancer cells from oxidative stress and compounds generating reactive oxygen species

**Figure 4:**
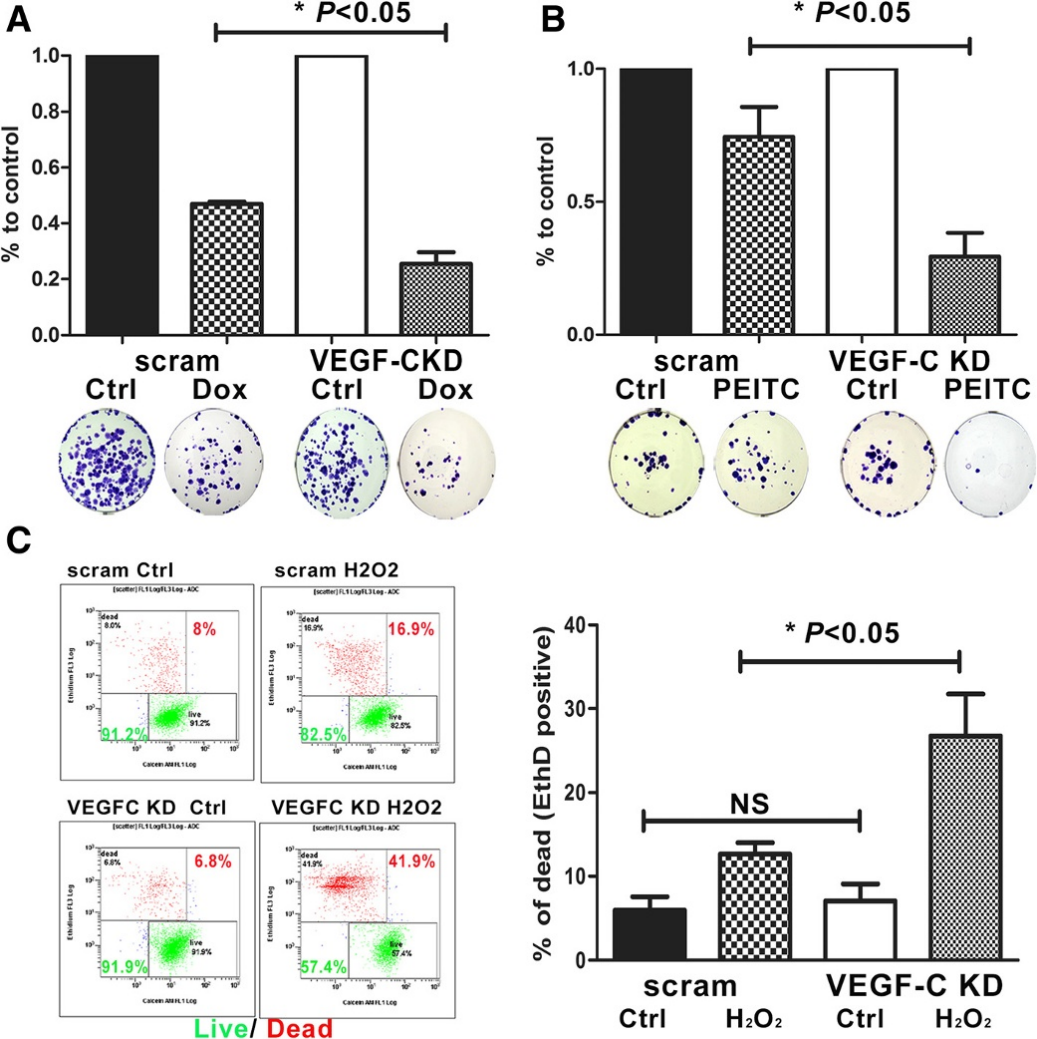
**Inhibition of vascular endothelial growth factor C sensitizes 66 cl4 mammary carcinoma cells to oxidative stress and to chemotherapeutic agents. (A)** Inhibition of vascular endothelial growth factor C (VEGF-C) in 66 cl4 cells decreases colony formation in the presence of doxorubicin (Dox). **(B)** Inhibition of VEGF-C in 66 cl4 cells decreases colony formation in the presence of phenethyl isothiocyanate (PEITC). Representative quantification (of three independent experiments) is shown for the number of colonies formed in 66 cl4-scram and VEGF-C KD1/KD2 cells (top). Representative pictures of colonies formed in 66 cl4-scram and VEGF-C KD cells treated with doxorubicin (3 μM) or PEITC (1 μM) or the corresponding vehicle control (Ctrl; bottom). **(C)** Representative flow data show the staining of live (calcein AM dye) and dead (ethidium homodimer (EthD) 1 dye) cell populations in 66 cl4-scram and VEGF-C KD cells treated with control (H_2_O) or H_2_O_2_ (1 mM) (left). Quantification of dead cells from 66 cl4-scram and 66 cl4-VEGF-C KD1/KD2 cells treated with control and put under oxidative stress (combination of three independent experiments).

Because VEGF-C expression correlates with gene sets that are enriched in TICs and upregulated in breast tumors after chemotherapy (Figure 1), we examined whether VEGF-C confers resistance to commonly used chemotherapeutic agents in breast cancer cells. Analysis of microarray data sets showed that breast cancer cell lines that are resistant to chemotherapeutic drugs [36],[37], specifically etoposide and doxorubicin, express higher levels of *VEGF C* than cell lines that are sensitive to these drugs (Additional file 5: Figure S5A). Indeed, we found that VEGF-C KD sensitizes 66 cl4 cells to doxorubicin and etoposide treatment (Additional file 5: Figure S5B). We validated these findings using a clonogenic assay and further tested the effect of another compound, PEITC, which is a natural compound from cruciferous vegetables that harbors both chemopreventive and chemotherapeutic activities and which has been shown to cause cancer cell death, specifically by disrupting antioxidant systems [3],[38],[39]. Both doxorubicin and PEITC treatment significantly decreased the ability of 66 cl4 cells to form colonies when VEGF-C was knocked down, as compared to scramble control KD cells (Figure 4A and B).

Chemotherapies are known to induce DNA damage in part by increasing cellular redox levels, and TICs are thought to have enhanced antioxidant pathways. Thus, we next investigated whether VEGF-C may protect breast cancer cells from oxidative stress. Induction of oxidative stress via treatment with a high concentration of H_2_O_2_ (1 mM) led to a significant increase in cell death and a decrease in cell viability when VEGF-C was knocked down in 66 cl4 cells (Figure 4C and Additional file 6: Figure S6). A similar result was observed in MDA-MB-231 breast cancer cells in which VEGF-C was knocked down (Additional file 7: Figure S7). These results suggest an important function of VEGF-C in protecting mammary carcinoma cells from oxidative stress or chemotherapy-induced cell death, implying that inhibition of VEGF-C may be a potential adjuvant therapy in combination with ROS-generating chemotherapeutic drugs in the treatment of breast cancers.

### Superoxide dismutase is regulated by VEGF-C

**Figure 5:**
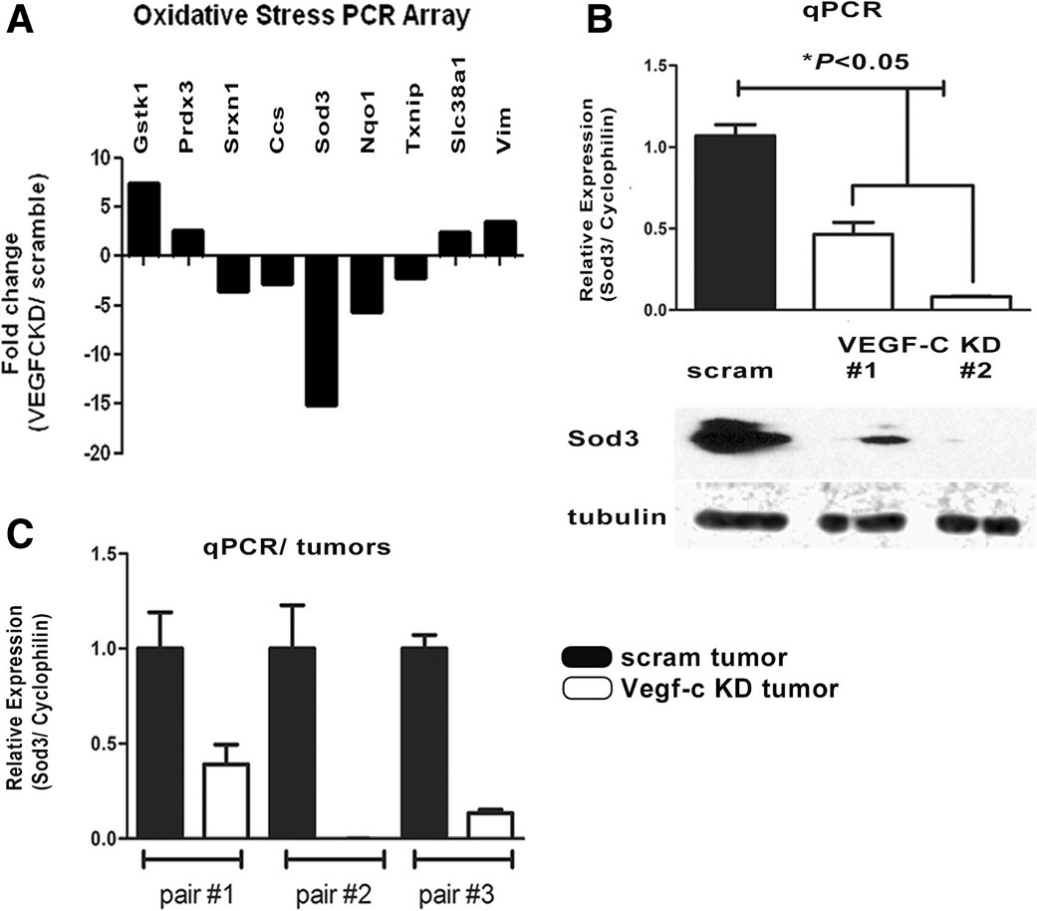
**Vascular endothelial growth factor C regulates Sod3 expression in 66 cl4 mammary carcinoma cells. (A)** Total RNA from 66 cl4-scramble and 66 cl4-VEGF-C KD2 cells was converted to cDNA and used to perform a mouse oxidative stress PCR array. Nine candidate genes of the eighty-four examined were identified in the PCR array with more than a twofold change in response to VEGF-C KD. **(B)** Sod3 mRNA expression was determined using a real-time PCR SYBR Green assay on 66 cl4-scram and 66 cl4-VEGF-C KD1 and KD2 cells (top). Western blot analysis of Sod3 expression in 66 cl4-scram and VEGF-C KD1 and KD2 cells (bottom). **(C)** Sod3 mRNA expression was determined by real-time PCR SYBR Green assay on three pairs of 66 cl4-scram and VEGF-C KD1 or KD2 tumors (each pair was derived from the same animal).

To elucidate the mechanism by which VEGF-C contributes to cell survival in the face of oxidative stress in breast cancer cells, we performed a quantitative PCR array to determine whether any genes related to oxidative stress were altered by VEGF-C. We identified 9 genes of the 84 examined that were regulated (up or down) twofold or more in response to VEGF-C KD (Figure 5A). Sod3 (which encodes extracellular Sod3 and plays a crucial role in scavenging superoxide) was found to have the highest and most consistent differences in expression between the 66c14-scramble and 66 cl4-VEGF-C KD cells (Figure 5B), and the regulation of Sod3 by VEGF-C was also confirmed in MDA-MB-231 cells (Additional file 8: Figure S8). We also examined the levels of Sod3 in 66 cl4-scram and VEGF-C KD tumors that developed in the left and right abdominal mammary fat pads of the same mice. Decreased Sod3 expression was detected in three different VEGF-C KD tumors compared to the corresponding scramble control tumors (Figure 5C). Together, our results demonstrate that VEGF-C regulates Sod3.

### Restoration of Sodexpression partially rescues phenotypes mediated by VEGF-C

**Figure 6:**
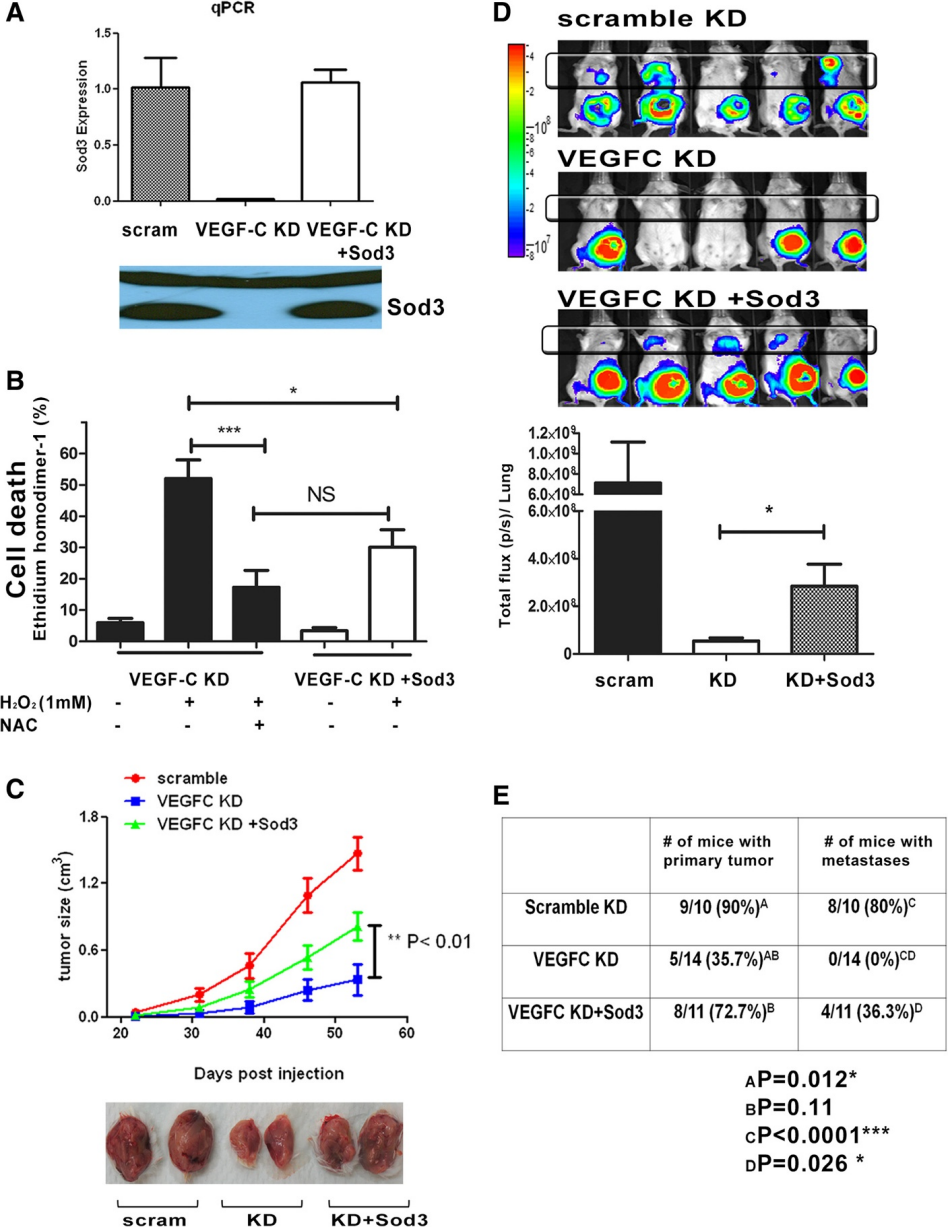
**Restoration of Sod3 in 66 cl4-VEGF-C KD cells partially rescues resistance to oxidative stress and tumor progression. (A)** Expression of Sod3 in 66 cl4-VEGF-C KD cells (KD2 in Figure 5). Empty vector was also introduced into 66 cl4-scram and 66 cl4-VEGF-C KD2 cells as a control. Sod3 expression was assessed in each cell line using a real-time PCR SYBR Green assay (top). Expression of secreted Sod3 in the media of 66 cl4-scram, 66 cl4-VEGF-C KD and 66 cl4-VEGF-C KD + Sod3 cells was measured by Western blot analysis (bottom). **(B)** Flow cytometry was performed to measure cell death induced by H_2_O_2_ under each condition shown. Three independent experiments were performed, and the data were combined for quantitation. **(C)** Cells from the 66 cl4-scram, VEGF-C KD and VEGF-C KD + Sod3 lines were injected into the fourth mammary fat pad of female BALB/c mice. Tumor growth in the mice was measured using calipers and calculated using the formula *V* = 1/2(W)(W)(L) (top). A representative picture of tumors from each group shows that restoration of Sod3 in VEGF-C KD cells partially rescues the size of tumors compared to scramble control tumors (bottom). **(D)** Representative *in vivo* image of 66 cl4-scram, VEGF-C KD and VEGF-C KD + Sod3 groups at day 60 after injection (top). Quantitation of bioluminescence imaging (in photons per second) emanating from the region surrounding the lungs (bottom). Mice that did not develop primary tumors were excluded from the quantitation. **(E)** Incidence of tumor formation and metastasis in the groups of mice injected with 66 cl4-scram, VEGF-C KD or VEGF-C KD + Sod3 cells. Restoration of Sod3 expression in the VEGF-C KD cells increased the number of mice that developed primary tumors and metastases, although not to the levels observed in the scramble control group. Fisher's exact test (two-sided) results indicated a significant increase in the number of mice that developed metastases when Sod3 expression was restored in the VEGF-C KD tumors.

To determine whether Sod3 mediates the ability of VEGF-C to act as an antioxidant, we reintroduced Sod3 into VEGF-C KD cells (using KD2 from Figure 5B) to levels similar to those observed in the scramble control cells (Figure 6A). In addition, the scramble control and VEGF-C KD cells were transfected with an empty vector to serve as a control. We confirmed the functionality of restored Sod3 in the VEGF-C KD, as its reintroduction rescues cell death induced by oxidative stress almost as efficiently as the antioxidant *N*-acetylcysteine (NAC) (Figure 6B). To determine whether restoration of Sod3 in the VEGF-C KD cells could rescue tumor progression, we injected luciferase labeled 66 cl4-scramble control, 66 cl4-VEGF-C KD and 66 cl4-VEGF-C Sod3 restored (VEGF-C KD + Sod3) cell lines into immunocompetent female BALB/c mice, and tumor growth and metastases were measured. We observed that reexpression of Sod3 in the VEGF-C KD cells restored their ability to grow and metastasize *in vivo* compared to VEGF-C KD cells (Figure 6C and D). In addition, when total numbers of mice with tumors and/or metastases were counted, we observed that VEGF-C KD resulted in a significant decrease in the number of mice that developed primary tumors and metastases in response to orthotopic injections and that restoration of Sod3 in VEGF-C KD cells trended toward rescuing the number of mice in which primary tumors formed and significantly rescued the number of mice that developed metastases (Figure 6E). Our results demonstrate a critical role for Sod3 downstream of VEGF-C in mediating tumor progression.

### Nrp2 regulates Sod3 expression and response of mammary carcinoma cells to oxidative stress

**Figure 7:**
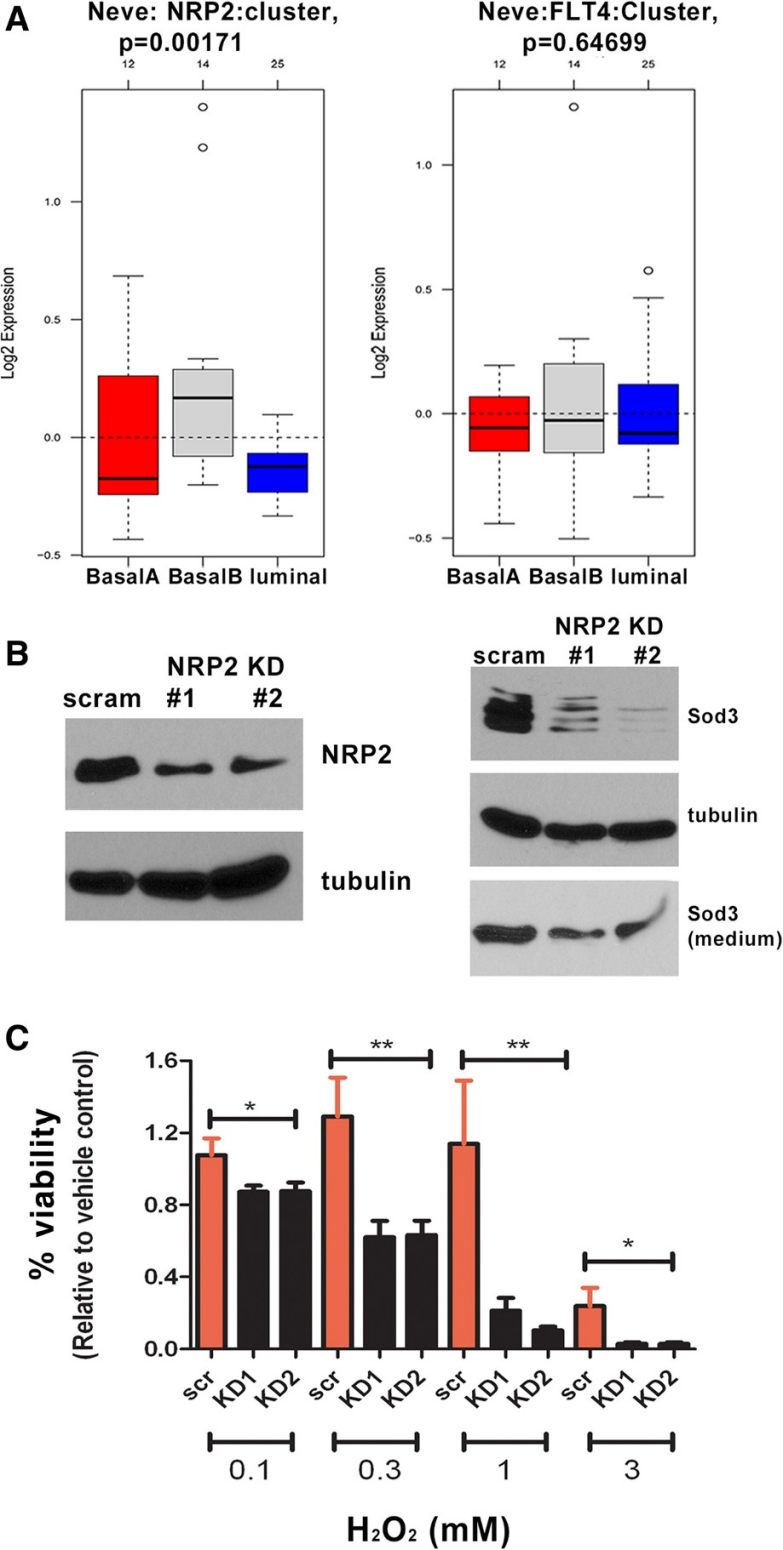
**Neuropilin 2-knockdown in 66 cl4 mouse mammary carcinoma cells decreases superoxide dismutase 3 expression and sensitizes cells to oxidative stress. (A)** *NRP2* and *VEGFR3* mRNA expression in human breast cancer cell lines. Box plots of *NRP2* (left) or *VEGFR3* (*FLT4*) (right) gene expression across 51 previously reported breast cancer cell lines grouped into basal A, basal B and luminal subgroups. *NRP2* and *VEGFR3* gene expression in human breast cancer cell lines was assessed using GOBO [21]. **(B)** Two different short-hairpin RNAs (shRNAs 1 and 2) were used to knock down neuropilin 2 (Nrp2) in 66 cl4 cells. Expression of Nrp2 in 66 cl4-scram and 66 cl4-Nrp2 knockdown (KD) cells was determined by Western blot analysis (left). Decreased expression of superoxide dismutase 3 (Sod3) was observed in 66 cl4-Nrp2 KD cells compared to 66 cl4-scram cells (right). Whole-cell lysates (top) or media (bottom) from 66 cl4-scram and 66 cl4-Nrp2 KD cells were collected for the detection of Sod3 by Western blotting. **(C)** Viability of 66 cl4-scram and 66 cl4-Nrp2 KD cells treated with increasing doses of H_2_O_2_. A CellTiter-Glo assay was used to measure viable cells. Three independent experiments were performed and combined for quantification.

Reexpression of VEGF-C-associated receptors on tumor cells has been observed, which suggests autocrine regulation of tumor cells by VEGF-C. Indeed, studies provide evidence that VEGF-C-related receptors mediate aggressive phenotypes of tumor cells [8],[14],[40]. To further investigate which of the known VEGF-C receptors may mediate the increase in Sod3 expression and the antioxidant phenotype, we first determined the expression of the receptors in 66 cl4 mammary carcinoma cells. Corroborating a previous finding [41], 66 cl4 cells primarily expressed Nrp2 and had undetectable levels of VEGF receptor 3 (VEGFR3) (Additional file 9: Figure S9). Examination of the Neve *et al*. cell line microarray data set [21],[22] revealed that *NRP2* expression tracks similarly to *VEGF C* expression, as both are expressed predominantly in basal B breast cancer cell lines (Figure 7A, left). In contrast, *VEGFR3* is not enriched in any specific subtype of breast cancer cell lines (Figure 7A, right). Thus, to determine whether NRP2 is the relevant receptor for VEGF-C in this system, we asked whether Nrp2 KD could recapitulate VEGF-C KD. Indeed, knockdown of Nrp2 in the 66 cl4 mammary carcinoma cell line (Figure 7B) led to a significant decrease in Sod3 expression compared to control KD cells (Figure 7B and Additional file 10: Figure S10). Importantly, knockdown of Nrp2 sensitized 66 cl4 mammary carcinoma cells to oxidative stress-induced cell death (Figure 7C), similar to what we observed with VEGF-C KD. The fact that Nrp2 (a known receptor for VEGF-C) loss phenocopies VEGF-C loss strongly suggests that VEGF-C acts through Nrp2 to mediate changes in Sod3 levels and the response to oxidative stress.

### VEGF C and SODare positively correlated in human cancers

**Figure 8:**
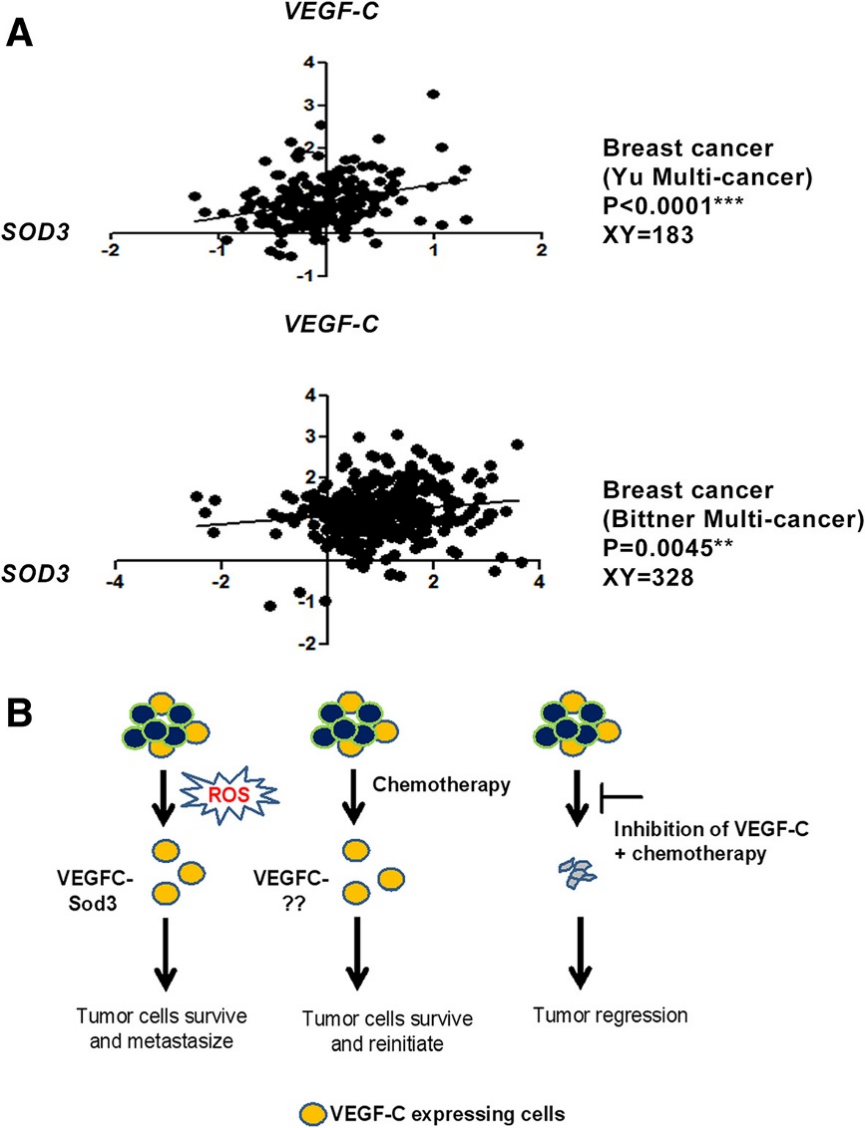
**Expression of** ***VEGFC*** **and** ***SOD3*** **in human cancers. (A)** *VEGFC* and *SOD3* expression values were retrieved from an Oncomine microarray data set [43] (as indicated in the figure) and were plotted by expression value. Statistical analysis was performed using Pearson r correlation (two-tailed). **(B)** Proposed model for the function of vascular endothelial growth factor C (VEGF-C) in breast cancer progression. Expression of VEGF-C in a subset of tumor cells confers the ability to resist oxidative stress generated during tumor growth, and this ability is partially mediated by Sod3. However, VEGF-C mediates other pathways that are important in conferring resistance to chemotherapies, thus contributing to tumor recurrence. Blocking VEGF-C signaling would therefore be expected to sensitize breast cancers to chemotherapies that induce oxidative stress, to contribute to a reduction in tumor-initiating cells (TICs) and to decrease lymphangiogenesis, thus likely improving survival and prevent recurrence.

To determine whether VEGF-C regulates SOD3 in human cancers, we examined whether their expression correlates in tumors. Interestingly, we found that expression of *VEGFC* and *SOD3* positively correlate not only in breast cancer (using two different human data sets) [42] (Bittner Multi-cancer data set, unpublished data, 1 January 2006) but also in kidney and cervical cancers (Bittner Multi-cancer data set) (Figure 8A and Additional file 11: Figure S11). Thus, these data further support our findings and suggest that the relationship between VEGF-C and Sod3 is relevant to human tumors.

## Discussion

In genomic studies, researchers have identified five major breast cancer intrinsic subtypes, including luminal A and B, HER2-positive, basal and claudin-low. Claudin-low breast cancers constitute about 7% to 14% of all molecular subtypes. These tumors show EMT gene expression profiles, enhanced cancer stem cell profiles and are typically less differentiated than the other subtypes of breast cancer [24]. Claudin-low breast cancers are highly aggressive, and the majority of the tumors lack hormone (ER and PR) and HER2 expression. Thus, patients harboring this subtype of breast cancer have a poorer prognosis, in part due to the lack of targeted therapies. In this study, we analyzed microarray data sets and demonstrate for the first time that *VEGF C* expression is high in the more aggressive subtypes of breast cancer (basal B and claudin-low), both in cell lines and in human tumors. Existing evidence suggests that lymphatic density and/or invasion is not more commonly observed in the more aggressive subtypes of breast cancer [28],[29],[44],[45], implying that high VEGF-C expression may confer alternate roles in mediating tumor aggressiveness in the claudin-low subtype. Because higher frequencies of TICs are found in these poorly differentiated types of breast cancers, we investigated whether VEGF-C correlates with a cancer stem cell signature in human breast cancer. In addition to analyzing how VEGF-C itself correlates with different tumor-related gene expression signatures, we created a *VEGF C*-13 gene signature and found that this gene set correlates with signatures which have been implicated in poor clinical outcome, including cancer stemness and chemoresistance, and that it correlates with a gene signature which is associated with shortened time to metastasis. These data imply that high VEGF-C expression in breast cancer may contribute to tumor progression by mediating a TIC-like phenotype, therapy resistance and metastasis.

Families of growth factors or cytokines, such as fibroblast growth factor or interleukin 6, respectively, are known to play a role in maintaining and/or expanding cancer stem-like populations through autocrine or paracrine signaling [46],[47]. The VEGF family has been studied extensively with respect to its role in the development of the vasculature and/or lymphatic system. In contrast, very few studies have implicated VEGF family members in the regulation of TIC properties. Recently, the results of some studies have pointed to a role for the receptors of VEGF family members in cancer stem-like properties through autocrine signaling pathways. For example, the VEGFR2 axis was shown to promote the viability and growth of glioma stem-like cells, and NRP2 has been implicated in breast tumor initiation [40],[48]. A previous study demonstrated that VEGF-C levels are enriched in TICs isolated from breast cancer lesions and from the MCF7 breast carcinoma cell line [49], and we extend these findings to demonstrate for the first time a *functional* role for VEGF-C in the regulation of breast TIC populations both *in vitro* and *in vivo*. Taken together with our finding that levels of VEGF-C are elevated in claudin-low breast cancers, our data suggest that high VEGF-C may be important in maintaining the TIC population in the claudin-low subtype of breast cancer, which is known to have prevalent TIC features.

Our study also provides evidence that VEGF-C mediates additional characteristics associated with TICs, such as modulating antioxidant responses and chemoresistance. Conventional radiation and therapeutic agents are known to cause DNA damage and apoptosis in tumors at least in part by generating oxidative stress. In a heterogeneous tumor, a small population of cells, the TIC population, is thought to enhance antiredox systems which allow them to survive after chemotherapy [5], thereby accounting for disease recurrence. Indeed, residual breast cancer cells isolated from patients who have received conventional therapy exhibit stem cell-like features and express increased antioxidant enzymes [4],[5],[31]. Because we observed that VEGF-C KD decreased TIC populations, we asked whether VEGF-C expression may also be involved in the response to oxidative stress in breast cancer cells. In support of our findings in breast cancer, VEGF-C was recently implicated in the response to ROS in prostate cancer cell lines [50]. Our data demonstrate that VEGF-C does protect breast cancer cells from ROS-induced cell death. Importantly, we identify, for the first time to our knowledge, an antioxidant factor—Sod3—as a downstream effector of VEGF-C. We show that Sod3 is at least in part responsible for the ability of VEGF-C to protect against ROS-induced cell death and to mediate breast tumor progression.

The superoxide dismutase family contains three members (Sod1, Sod2 and Sod3) that protect cells and/or tissues against intracellular or extracellular ROS damage. However, the role of Sod family members has not been well studied in cancer, and where it has been studied, the roles are controversial. For example, studies exist that show a protective role for Sod3 against chemical or hormone-induced tumor formation [51],[52]. In contrast, other studies have demonstrated that expression of Sod2 maintains the metabolic activity of cancer cells when they are detached from the extracellular matrix and that it increases tumor promotional nuclear factor (NFκB) signaling [53],[54]. In our present study, we demonstrate a tumor promotional effect of Sod3 downstream of VEGF-C in that restoration of Sod3 in the VEGF-C KD cells not only partially rescues tumor growth but also rescues metastasis. However, since Sod3 is not sufficient to fully rescue the *in vivo* tumor growth and/or metastasis in response to VEGF-C KD (Figure 6), nor is it sufficient to rescue chemoresistance after VEGF-C KD *in vitro* (Additional file 12: Figure S12), other pathways and/or factors downstream of VEGF-C must be required to fully restore these phenotypes. Given the results of our ROS array, it is possible that VEGF-C stimulates antioxidant effects via regulating several redox-related enzymes and that, though Sod3 is a major regulator of the ROS response downstream of VEGF-C, it is not the only one. It is also possible that VEGF-C confers TIC-related chemoresistance via mediating additional signaling pathways (such as Wnt/β-catenin, Notch or NFκB signaling) [55]. Furthermore, because VEGF-C can also promote metastasis through its ability to mediate lymphangiogenesis, and because VEGF-C expression in tumor cells can affect the infiltration of immune cells [56],[57], multiple functions of VEGF-C are likely required to mediate its full effects on tumor progression and metastasis.

On the basis of our results, we propose a novel model, shown in Figure 8B. VEGF-C expression in a small population of TICs is important in performing multiple functions during tumor progression. In part, VEGF-C promotes tumor progression via regulating Sod3, which can eliminate the excessive oxidative species generated when tumor cells are rapidly growing. However, other pathways downstream of VEGF-C, not limited to Sod3 regulation, are involved in the ability of cells to survive chemotherapy. Thus, blocking VEGF-C may be useful in combination with chemotherapeutic drugs (especially with those known to induce ROS) to increase treatment efficacy, because inhibition of VEGF-C may decrease the TIC population, sensitize cells to oxidative stress–induced cell death and affect the tumor microenvironment by decreasing lymphangiogenesis.

## Conclusions

We have uncovered a novel mechanism for the vascular endothelial growth factor, VEGF-C, in regulating tumor rather than lymphatic endothelial cells. We show, for the first time to our knowledge, that VEGF-C is highly expressed in the aggressive claudin-low subtype of breast cancer, and we further demonstrate that VEGF-C expression correlates with TIC and chemoresistant signatures. Our data demonstrate that high VEGF-C expression leads to an increase in the TIC population, an altered cellular response to ROS and an increase in resistance to chemotherapy, all likely contributing to its ability to enhance tumor progression. We uncover a novel mechanism by which VEGF-C regulates the response to oxidative stress, via regulation of Sod3 expression. Importantly, Sod3 downstream of VEGF-C is required for tumor growth and metastasis in the mammary orthotopic xenograft model, providing evidence that VEGF-C mediates breast cancer metastasis in part through regulating ROS. Our results suggest that inhibition of VEGF-C may suppress TIC-like phenotypes and sensitize breast tumors to chemotherapy in the claudin-low subtype, for which few targeted therapies currently exist.

## Additional files

## Electronic supplementary material

Additional file 1: Figure S1.: Generation of the *VEGFC*-13 gene signature and the expression of *VEGFC*-13 gene signature in breast tumors. **(A)** Scatterplot of Pearson's correlation values for *VEGFC* and all genes within the UNC35 cell line database and UNC288 tumor database. Red box denotes 13 genes with Pearson's correlations >0.5 with *VEGFC* in both data sets. The *VEGFC* gene signature was identified by utilizing two data sets generated from Agilent two-color gene expression arrays (Agilent Technologies, Santa Clara, CA, USA). For the human breast tumor data set, 288 tumors representing all of the intrinsic subtypes were used (UNC288). For the human breast cancer cell line data set, 35 distinct human breast cancer cell lines (UNC35), also representing each subtype, were utilized [33],[34]. For both data sets, data were retrieved from the UNC MicroArray Database. To identify Pearson's correlation values, genes were median-centered, and all genes that were strongly correlated with *VEGFC* (>0.5) across all samples in both data sets were selected. **(B)** Table of genes correlated with *VEGFC* expression in both the UNC35 cell line database and the UNC288 tumor database. **(C)** and **(D)** Box-and-whisker plots show the expression of the *VEGFC* signature (VEGFC-13) in the UNC 337 and UNC855 tumor data sets. (PDF 161 KB)

Additional file 2: Figure S2.: Expression of *VEGFC* in attached parental cells or tumorspheres. MDA-MB-468, MDA-MB-231 and T47D human breast cancer cells were grown under attached conditions or formed by growing cells in serum-free suspension conditions. Real-time PCR was performed, and *VEGFC* relative expression was determined after normalization to *cyclophilin* gene expression in the cells. (PDF 66 KB)

Additional file 3: Figure S3.: Expression of *VEGFC* in 66 cl4-scramble and 66 cl4-VEGF-C-knockdown cells. *VEGFC* gene expression was detected by real-time PCR using the TaqMan assay. Two different shRNAs against *VEGFC* were delivered to the 66 cl4 cells, and stable knockdown cells were selected using puromycin. (PDF 43 KB)

Additional file 4: Figure S4.: VEGF-C is efficiently knocked down in MDA-MB-231 breast cancer cells. **(A)** Expression of *VEGFC* in the human MDA-MB-231 breast cancer cell line. Real-time PCR (TaqMan assay) was performed to determine relative expression of *VEGFC* in MDA-MB-231 compared to MCF7 or human dermal lymphatic endothelial cells (HDLECs). **(B)** Expression of VEGF-C in MDA-MB-231 control KD (NS) and two VEGF-C knockdown cells determined by Western blot analysis. β-actin was used as a loading control. (PDF 57 KB)

Additional file 5: Figure S5.: VEGF-C knockdown sensitizes 66 cl4 mammary carcinoma cells to chemotherapeutic agents. **(A)** Expression of *VEGFC* mRNA levels in breast cancer cell lines that are sensitive (including intermediate levels of sensitivity) or resistant to etoposide or doxorubicin were retrieved from the Garnett cell line and Györffy cell line data sets in Oncomine [36],[37]. **(B)** 66 cl4-scram and VEGF-C KD cell viability in response to different doses of etoposide or doxorubicin measured by CellTiter-Glo assay. Data from two VEGF-C KD cells were combined for quantification. Three independent experiments were performed. (PDF 92 KB)

Additional file 6: Figure S6.: Viability of 66 cl4-scram and 66 cl4-VEGF-C KD cells treated with increasing doses of H_2_O_2_. Luciferase activity of the cells was measured using *in vivo* imaging as an indicator of cell viability. As shown by quantifying the luciferase signal, VEGF-C KD sensitizes cells to H_2_O_2_-induced cell death, and cell viability can be restored by cotreatment with NAC, a strong antioxidant. (PDF 75 KB)

Additional file 7: Figure S7.: Viability of MDA-MB-231 control KD and two VEGF-C KD cells treated with increasing doses of H_2_O_2_. Cell viability was measured using the CellTiter-Glo assay, a luminescent detection of ATP in viable cells. Two independent experiments were performed on control cell lines and two different VEGF-C KD cell lines. (PDF 23 KB)

Additional file 8: Figure S8.: VEGF-C regulates SOD3 expression in MDA-MB-231 breast cancer cells. Sod3 protein expression was determined by Western blot analysis in MDA-MB-231 scram control cells and two VEGF-C KD cell lines. β-actin was used as a loading control. (PDF 45 KB)

Additional file 9: Figure S9.: Expression of VEGF-C receptors in 66 cl4 mammary carcinoma cells. Real-time PCR analysis was performed to determine the relative expression of *VEGFR3* and *NRP2* in 66 cl4 cells. NMuMG cells were used as a positive control for the expression of *VEGFR3*. 67NR is isogenic to 66 cl4, but is nonmetastatic (whereas 66 cl4 is metastatic). Expression of *VEGFR3* and *NRP2* was determined and plotted after normalization to cyclophilin expression (*Ppib*) in the cells. (PDF 157 KB)

Additional file 10: Figure S10.: *Sod3* mRNA expression in 66 cl4-Nrp2-knockdown cells. Real-time PCR analysis was performed to determine the relative expression of *Sod3* in 66 cl4 control and Nrp2 KD cells. Expression of *Sod3* was determined and plotted after normalization to cyclophilin (*Ppib*) expression in the cells. (PDF 52 KB)

Additional file 11: Figure S11.: Expression of *VEGFC* and *SOD3* in human cancers. *VEGFC* and *SOD3* expression values were retrieved from an Oncomine microarray data set (Bittner Multi-cancer data set) and were plotted by expression value. Statistical analysis was performed using Pearson r correlation (two-tailed). (PDF 48 KB)

Additional file 12: Figure S12.: Restoration of Sod3 in VEGF-C KD cells is not sufficient to rescue the ability of 66 cl4 cells to resist doxorubicin and PEITC-induced cell death. Viability of 66 cl4-scram, VEGF-C KD and VEGF-C KD + Sod3 cells treated with doxorubicin and PEITC as measured using *in vivo* imaging for luciferase activity as an indicator of cell viability. (PDF 110 KB)

Below are the links to the authors’ original submitted files for images.

Authors’ original file for figure 1

Authors’ original file for figure 2

Authors’ original file for figure 3

Authors’ original file for figure 4

Authors’ original file for figure 5

Authors’ original file for figure 6

Authors’ original file for figure 7

Authors’ original file for figure 8

## Abbreviations

ALDH: Aldehyde dehydrogenase
KD: Knockdown
Nrp2: Neuropilin 2
ROS: Reactive oxygen species
Sod3: Superoxide dismutase 3
TIC: Tumor-initiating cell
VEGF-C: Vascular endothelial growth factor C
