# Supplementary material for: Vascular endothelial growth factor C promotes breast cancer progression via a novel antioxidant mechanism that involves regulation of superoxide dismutase 3
Source: Breast Cancer Res. 2014 Oct 30;16:462. doi: 10.1186/s13058-014-0462-2 (PMC4303136; doi:10.1186/s13058-014-0462-2)

A

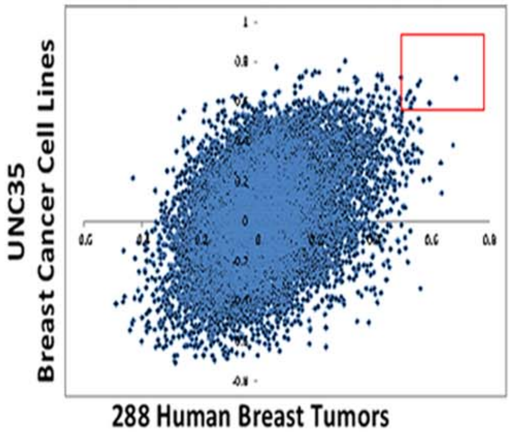

C

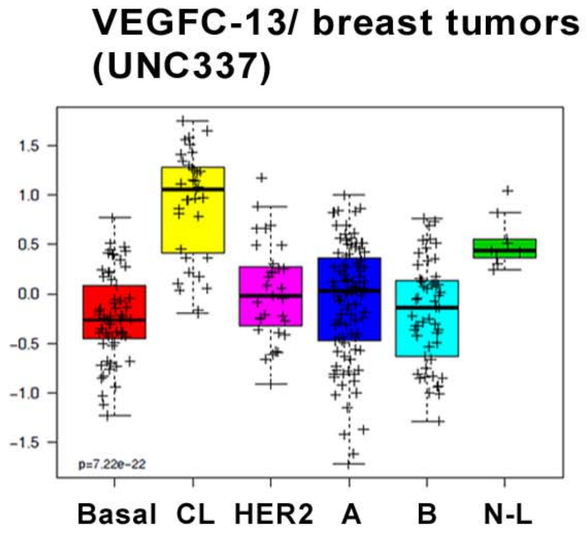

B

LocusLink	NAME	Pearson Correlation Value breast tumors	Pearson Correlation Value Breast Cancer Lines (Prat 2013)
7424	VEGFC	1	1
118429	ANTXR2	0.686901931	0.716101984
11167	FSTL1	0.593805958	0.591376067
25900	IFFO1	0.561215432	0.700377915
2200	FBN1	0.55669635	0.512446753
3678	ITGA5	0.5545269	0.681352674
53918	PELO	0.541157861	0.613634558
7431	VIM	0.530788033	0.591160764
25945	PVRL3	0.530435937	0.566938694
6695	SPOCK1	0.526673482	0.64569315
857	CAV1	0.52603232	0.569481686
8406	SRPX	0.518137766	0.62840912
29940	DSE	0.513749061	0.611208192

D

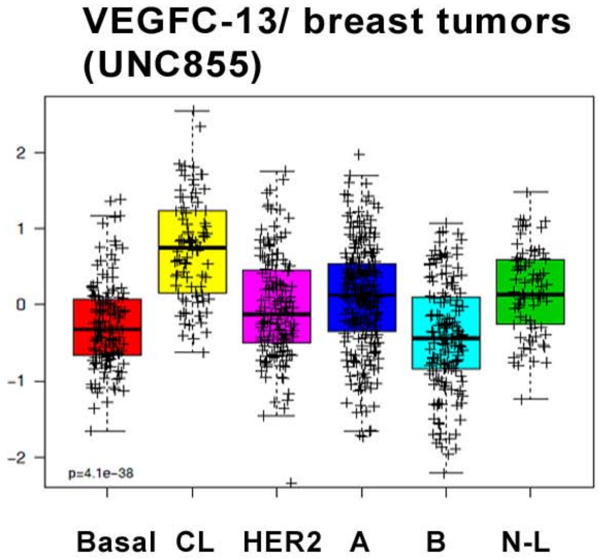

Supplement: Supplementary file 1 — Additional file 1: Figure S1.: Generation of the VEGFC-13 gene signature and the expression of VEGFC-13 gene signature in breast tumors. (A) Scatterplot of Pearson's correlation values for VEGFC and all genes within the UNC35 cell line database and UNC288 tumor database. Red box denotes 13 genes with Pearson's correlations >0.5 with VEGFC in both data sets. The VEGFC gene signature was identified by utilizing two data sets generated from Agilent two-color gene expression arrays (Agilent Technologies, Santa Clara, CA, USA). For the human breast tumor data set, 288 tumors representing all of the intrinsic subtypes were used (UNC288). For the human breast cancer cell line data set, 35 distinct human breast cancer cell lines (UNC35), also representing each subtype, were utilized [33],[34]. For both data sets, data were retrieved from the UNC MicroArray Database. To identify Pearson's correlation values, genes were median-centered, and all genes that were strongly correlated with VEGFC (>0.5) across all samples in both data sets were selected. (B) Table of genes correlated with VEGFC expression in both the UNC35 cell line database and the UNC288 tumor database. (C) and (D) Box-and-whisker plots show the expression of the VEGFC signature (VEGFC-13) in the UNC 337 and UNC855 tumor data sets. (PDF 161 KB) [file 13058_2014_462_MOESM1_ESM.pdf]
