# Supplementary figures and images for: Vascular endothelial growth factor C promotes breast cancer progression via a novel antioxidant mechanism that involves regulation of superoxide dismutase 3
Source: Breast Cancer Res. 2014 Oct 30;16:462. doi: 10.1186/s13058-014-0462-2 (PMC4303136; doi:10.1186/s13058-014-0462-2)

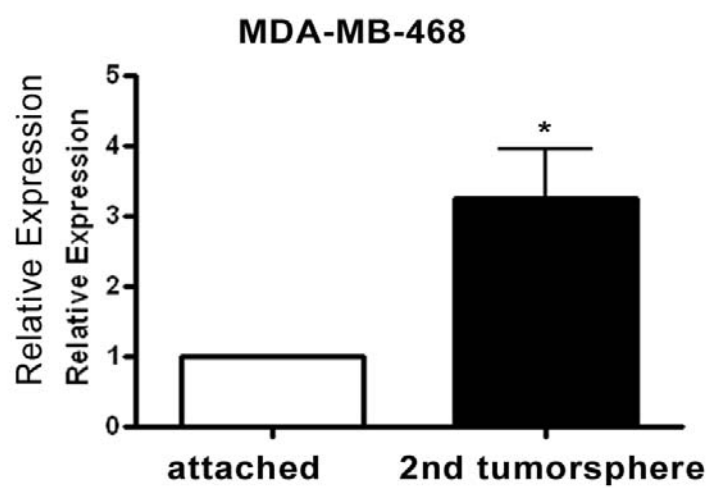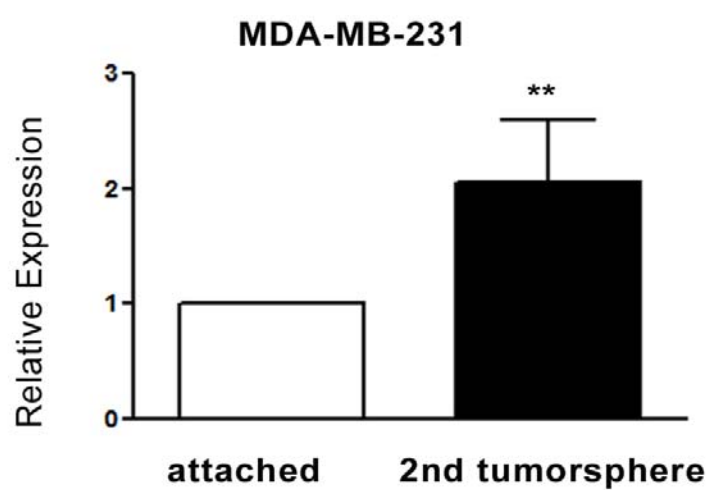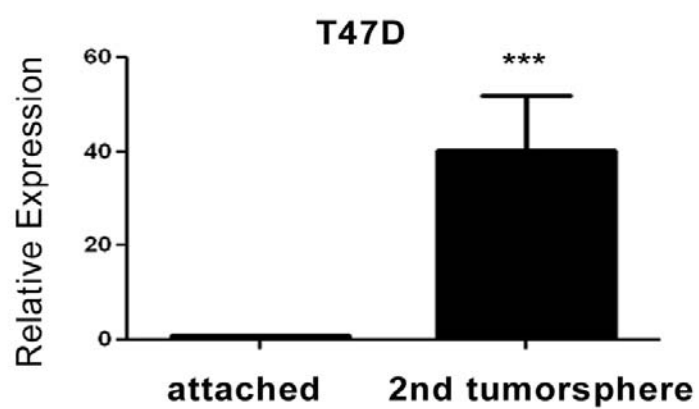

Supplement: Supplementary file 2 — Additional file 2: Figure S2.: Expression of VEGFC in attached parental cells or tumorspheres. MDA-MB-468, MDA-MB-231 and T47D human breast cancer cells were grown under attached conditions or formed by growing cells in serum-free suspension conditions. Real-time PCR was performed, and VEGFC relative expression was determined after normalization to cyclophilin gene expression in the cells. (PDF 66 KB) [file 13058_2014_462_MOESM2_ESM.pdf]

66cl4

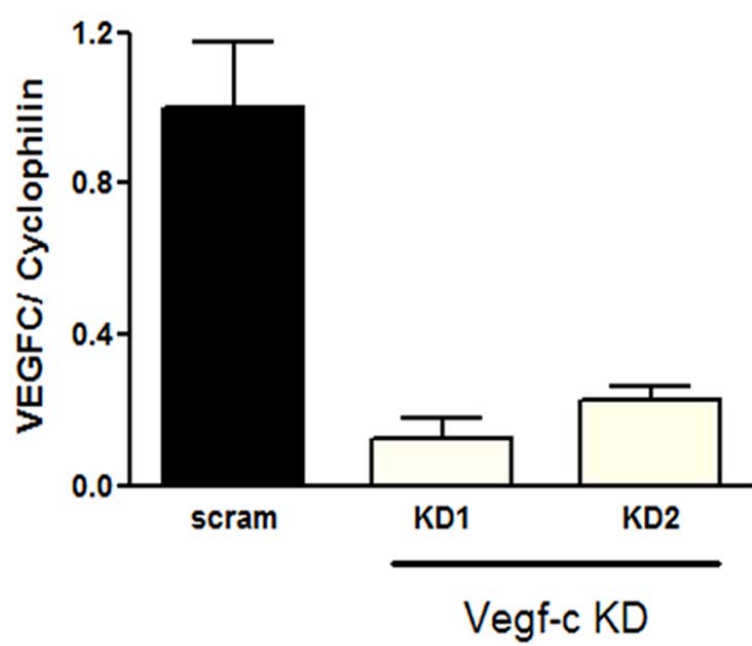

Supplement: Supplementary file 3 — Additional file 3: Figure S3.: Expression of VEGFC in 66 cl4-scramble and 66 cl4-VEGF-C-knockdown cells. VEGFC gene expression was detected by real-time PCR using the TaqMan assay. Two different shRNAs against VEGFC were delivered to the 66 cl4 cells, and stable knockdown cells were selected using puromycin. (PDF 43 KB) [file 13058_2014_462_MOESM3_ESM.pdf]

**A**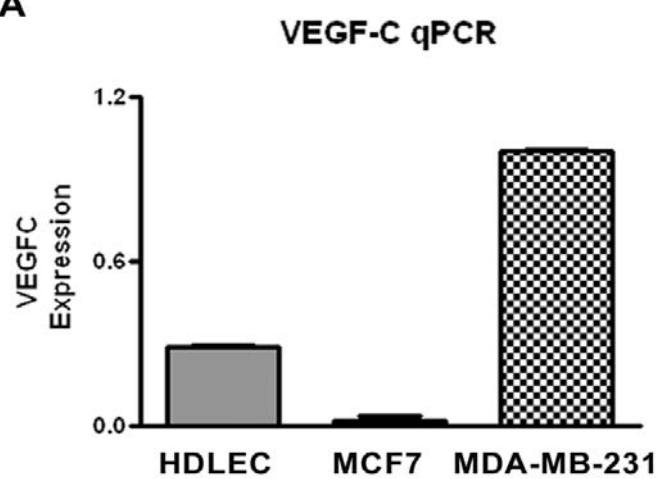**B**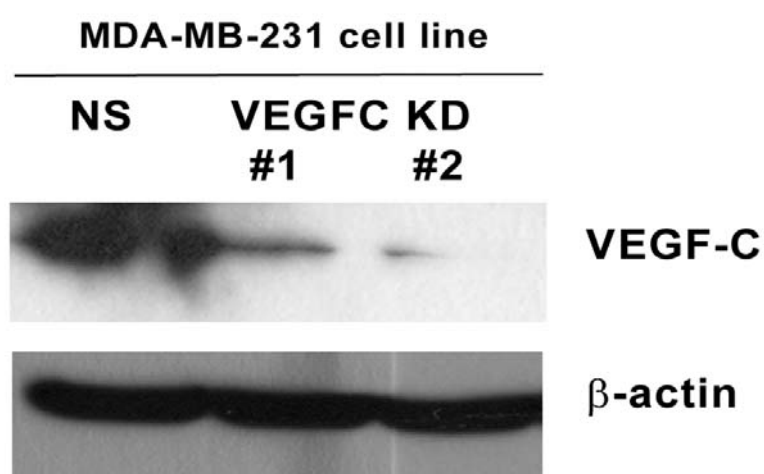

Supplement: Supplementary file 4 — Additional file 4: Figure S4.: VEGF-C is efficiently knocked down in MDA-MB-231 breast cancer cells. (A) Expression of VEGFC in the human MDA-MB-231 breast cancer cell line. Real-time PCR (TaqMan assay) was performed to determine relative expression of VEGFC in MDA-MB-231 compared to MCF7 or human dermal lymphatic endothelial cells (HDLECs). (B) Expression of VEGF-C in MDA-MB-231 control KD (NS) and two VEGF-C knockdown cells determined by Western blot analysis. β-actin was used as a loading control. (PDF 57 KB) [file 13058_2014_462_MOESM4_ESM.pdf]

**A**

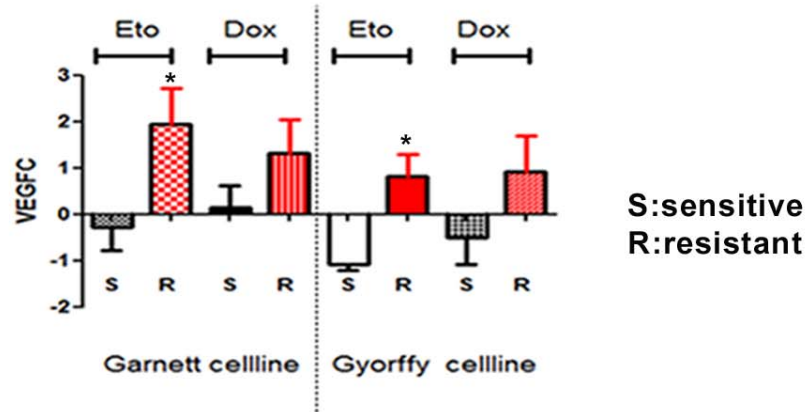

**B**

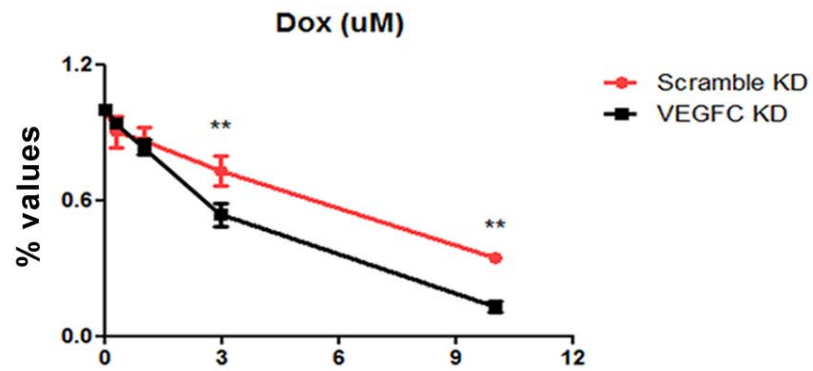

Doxorubicin (uM)

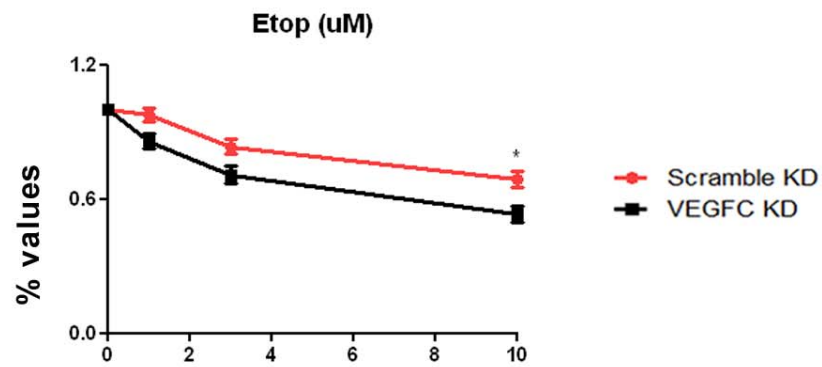

Etoposide (uM)

Supplement: Supplementary file 5 — Additional file 5: Figure S5.: VEGF-C knockdown sensitizes 66 cl4 mammary carcinoma cells to chemotherapeutic agents. (A) Expression of VEGFC mRNA levels in breast cancer cell lines that are sensitive (including intermediate levels of sensitivity) or resistant to etoposide or doxorubicin were retrieved from the Garnett cell line and Györffy cell line data sets in Oncomine [36],[37]. (B) 66 cl4-scram and VEGF-C KD cell viability in response to different doses of etoposide or doxorubicin measured by CellTiter-Glo assay. Data from two VEGF-C KD cells were combined for quantification. Three independent experiments were performed. (PDF 92 KB) [file 13058_2014_462_MOESM5_ESM.pdf]

## H2O2

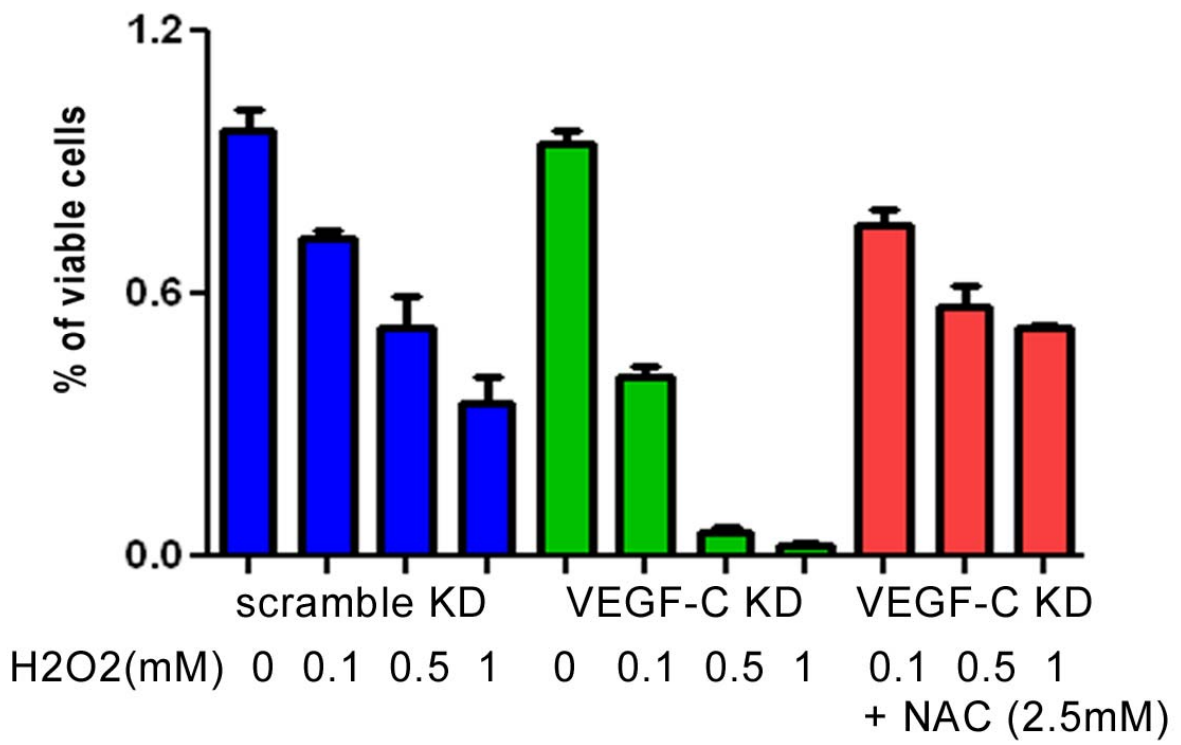

Supplement: Supplementary file 6 — Additional file 6: Figure S6.: Viability of 66 cl4-scram and 66 cl4-VEGF-C KD cells treated with increasing doses of H2O2. Luciferase activity of the cells was measured using in vivo imaging as an indicator of cell viability. As shown by quantifying the luciferase signal, VEGF-C KD sensitizes cells to H2O2-induced cell death, and cell viability can be restored by cotreatment with NAC, a strong antioxidant. (PDF 75 KB) [file 13058_2014_462_MOESM6_ESM.pdf]

## MDA-MB-231

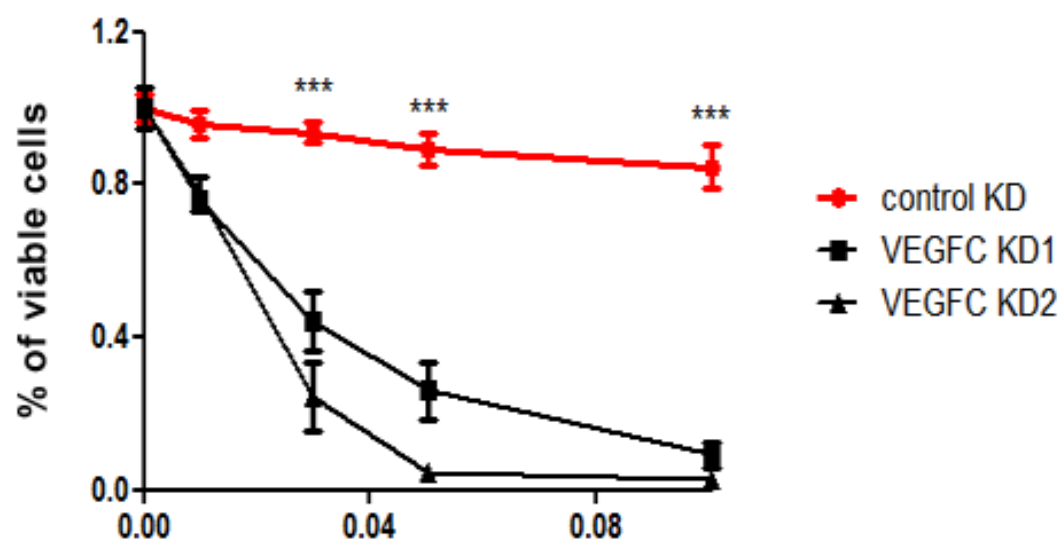

Supplement: Supplementary file 7 — Additional file 7: Figure S7.: Viability of MDA-MB-231 control KD and two VEGF-C KD cells treated with increasing doses of H2O2. Cell viability was measured using the CellTiter-Glo assay, a luminescent detection of ATP in viable cells. Two independent experiments were performed on control cell lines and two different VEGF-C KD cell lines. (PDF 23 KB) [file 13058_2014_462_MOESM7_ESM.pdf]

## MDA-MB-231

---

NS

VEGFC KD  
#1 #2

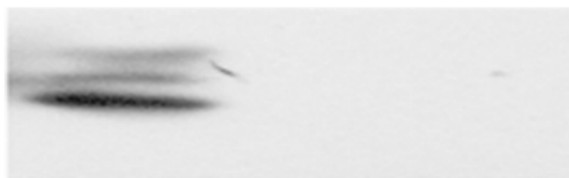

Sod3

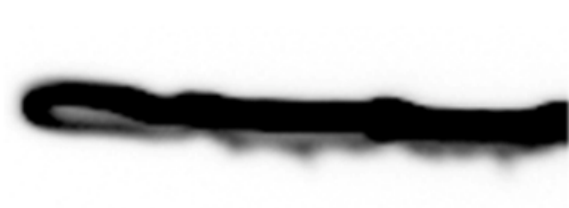

$\beta$ -actin

Supplement: Supplementary file 8 — Additional file 8: Figure S8.: VEGF-C regulates SOD3 expression in MDA-MB-231 breast cancer cells. Sod3 protein expression was determined by Western blot analysis in MDA-MB-231 scram control cells and two VEGF-C KD cell lines. β-actin was used as a loading control. (PDF 45 KB) [file 13058_2014_462_MOESM8_ESM.pdf]

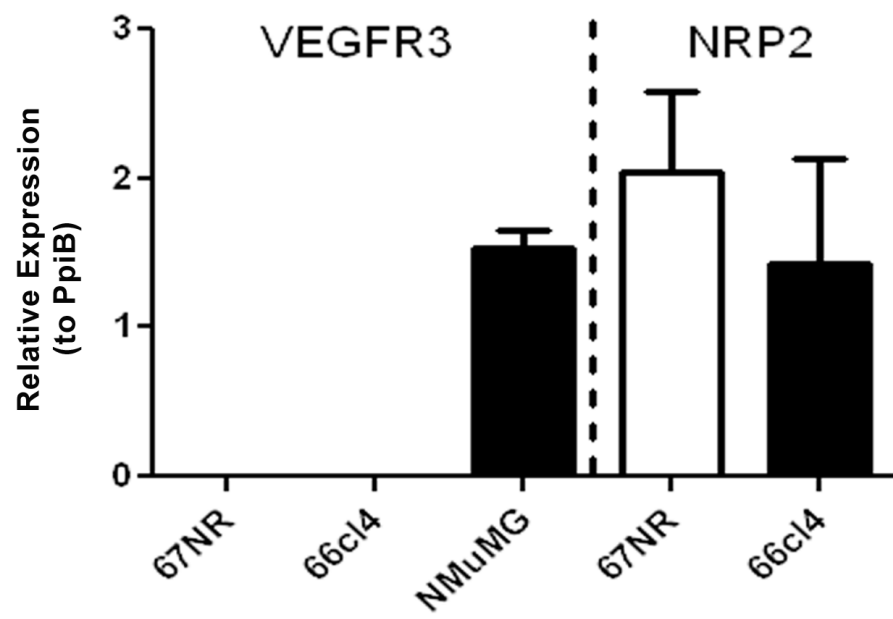

Supplement: Supplementary file 9 — Additional file 9: Figure S9.: Expression of VEGF-C receptors in 66 cl4 mammary carcinoma cells. Real-time PCR analysis was performed to determine the relative expression of VEGFR3 and NRP2 in 66 cl4 cells. NMuMG cells were used as a positive control for the expression of VEGFR3. 67NR is isogenic to 66 cl4, but is nonmetastatic (whereas 66 cl4 is metastatic). Expression of VEGFR3 and NRP2 was determined and plotted after normalization to cyclophilin expression (Ppib) in the cells. (PDF 157 KB) [file 13058_2014_462_MOESM9_ESM.pdf]

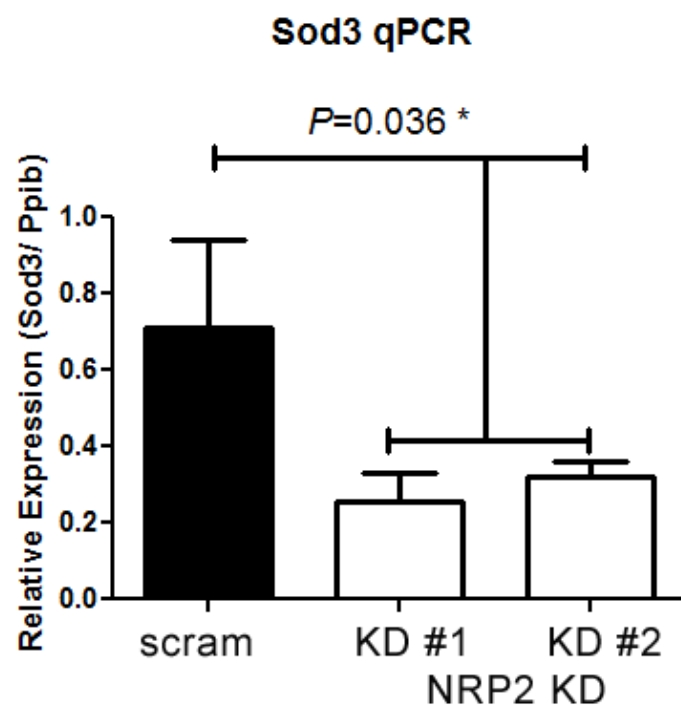

Supplement: Supplementary file 10 — Additional file 10: Figure S10.: Sod3 mRNA expression in 66 cl4-Nrp2-knockdown cells. Real-time PCR analysis was performed to determine the relative expression of Sod3 in 66 cl4 control and Nrp2 KD cells. Expression of Sod3 was determined and plotted after normalization to cyclophilin (Ppib) expression in the cells. (PDF 52 KB) [file 13058_2014_462_MOESM10_ESM.pdf]

Bittner Multi-cancer  
Kidney Cancer  
 $P=0.0014$  \*\* XY=254

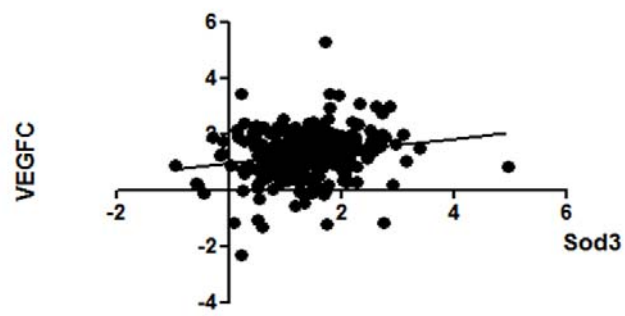

Bittner Multi-cancer  
Cervial Cancer  
 $P=0.0467$  \* XY=35

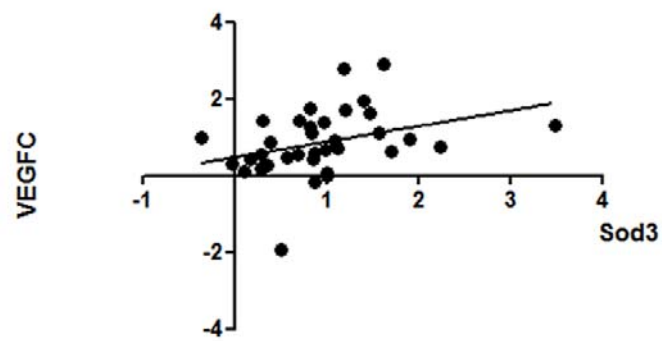

Supplement: Supplementary file 11 — Additional file 11: Figure S11.: Expression of VEGFC and SOD3 in human cancers. VEGFC and SOD3 expression values were retrieved from an Oncomine microarray data set (Bittner Multi-cancer data set) and were plotted by expression value. Statistical analysis was performed using Pearson r correlation (two-tailed). (PDF 48 KB) [file 13058_2014_462_MOESM11_ESM.pdf]

Doxorubicin (uM)

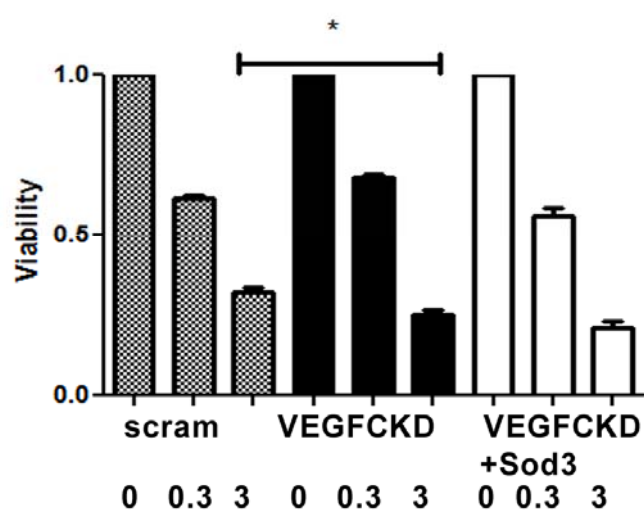

PEITC (uM)

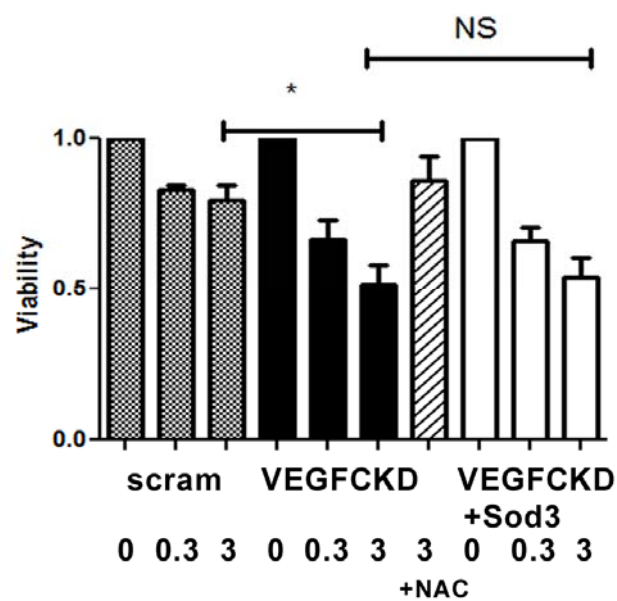

Supplement: Supplementary file 12 — Additional file 12: Figure S12.: Restoration of Sod3 in VEGF-C KD cells is not sufficient to rescue the ability of 66 cl4 cells to resist doxorubicin and PEITC-induced cell death. Viability of 66 cl4-scram, VEGF-C KD and VEGF-C KD + Sod3 cells treated with doxorubicin and PEITC as measured using in vivo imaging for luciferase activity as an indicator of cell viability. (PDF 110 KB) [file 13058_2014_462_MOESM12_ESM.pdf]

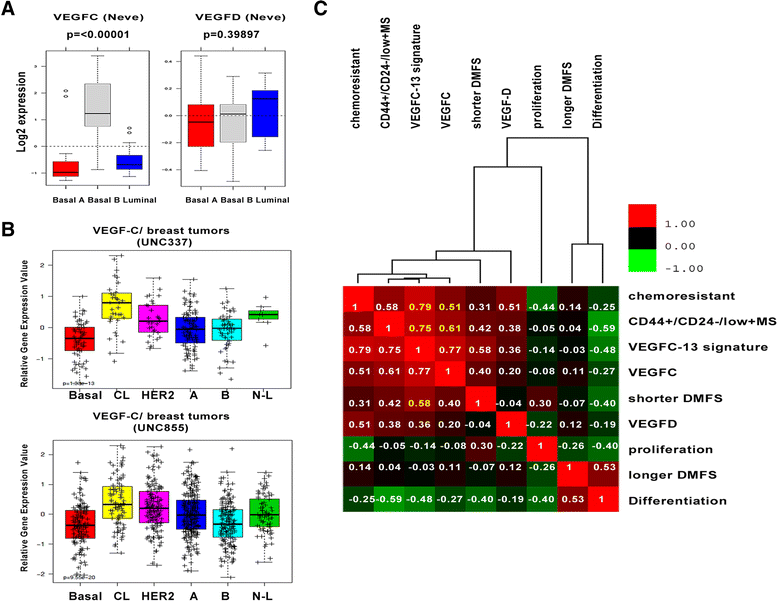

Supplement: Supplementary file 13 — Authors’ original file for figure 1 [file 13058_2014_462_MOESM13_ESM.gif]

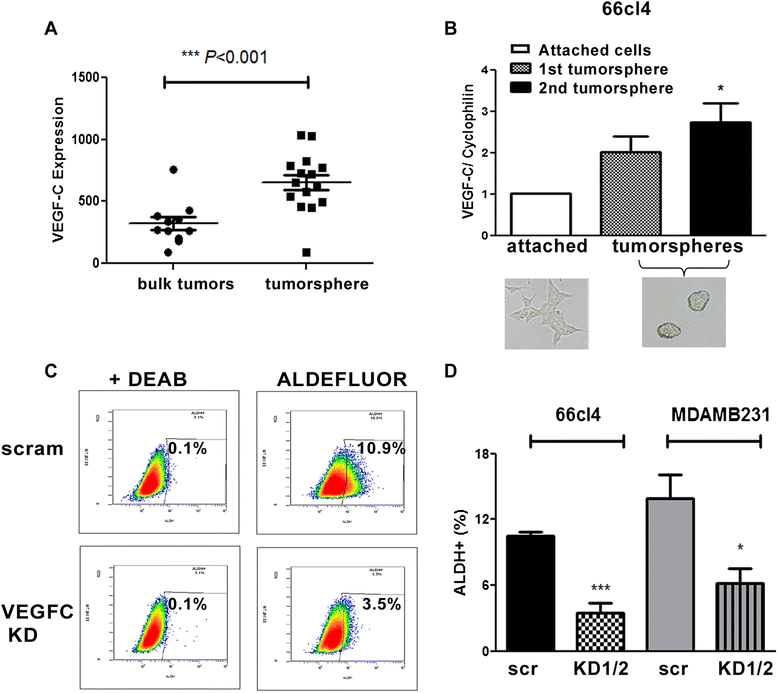

Supplement: Supplementary file 14 — Authors’ original file for figure 2 [file 13058_2014_462_MOESM14_ESM.gif]

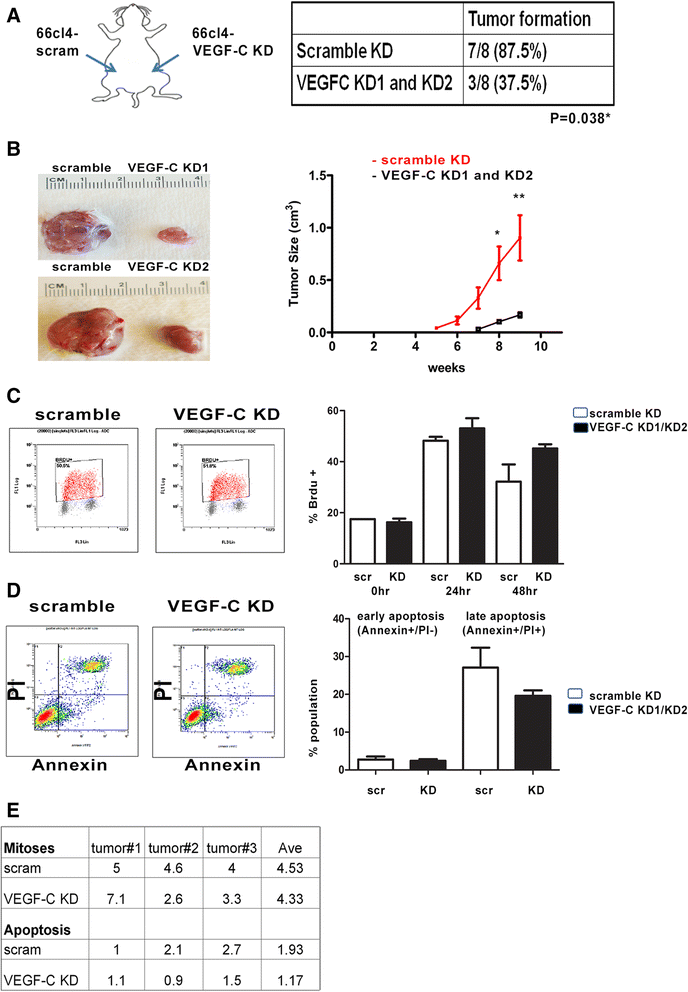

Supplement: Supplementary file 15 — Authors’ original file for figure 3 [file 13058_2014_462_MOESM15_ESM.gif]

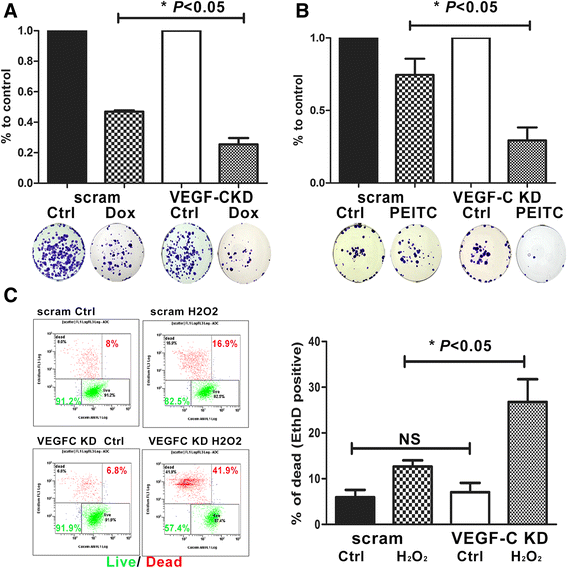

Supplement: Supplementary file 16 — Authors’ original file for figure 4 [file 13058_2014_462_MOESM16_ESM.gif]

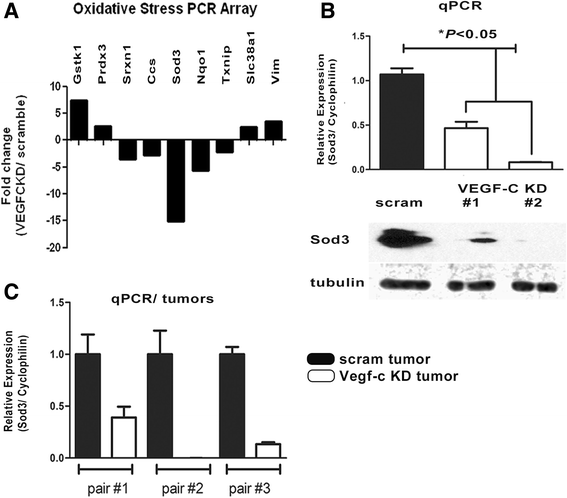

Supplement: Supplementary file 17 — Authors’ original file for figure 5 [file 13058_2014_462_MOESM17_ESM.gif]

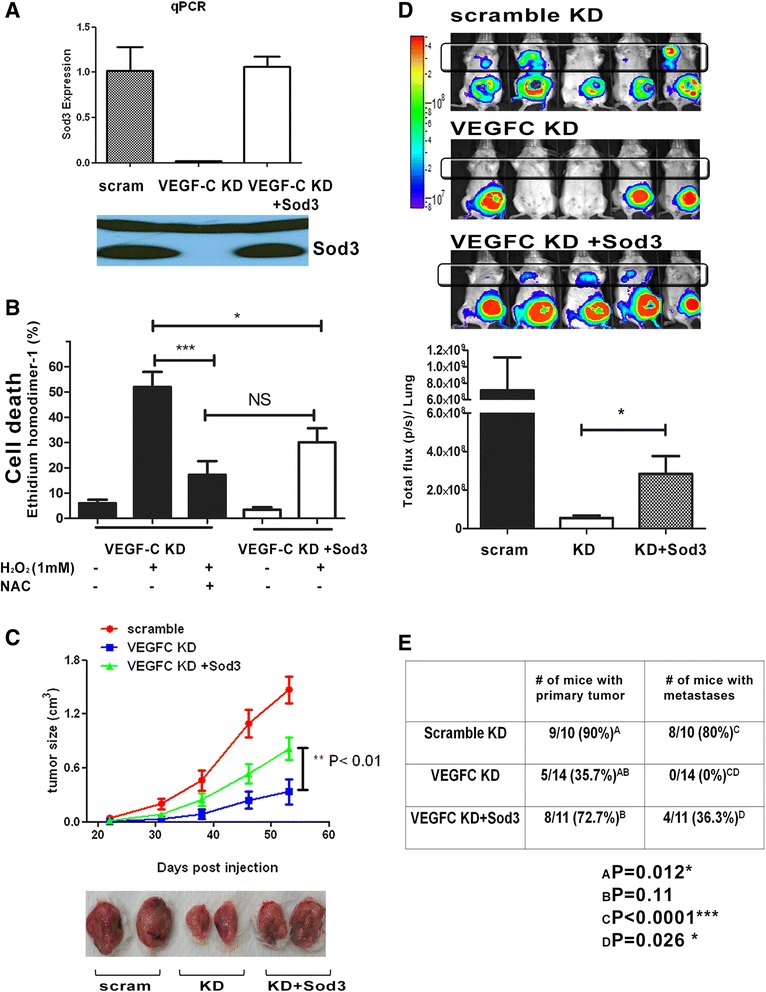

Supplement: Supplementary file 18 — Authors’ original file for figure 6 [file 13058_2014_462_MOESM18_ESM.gif]

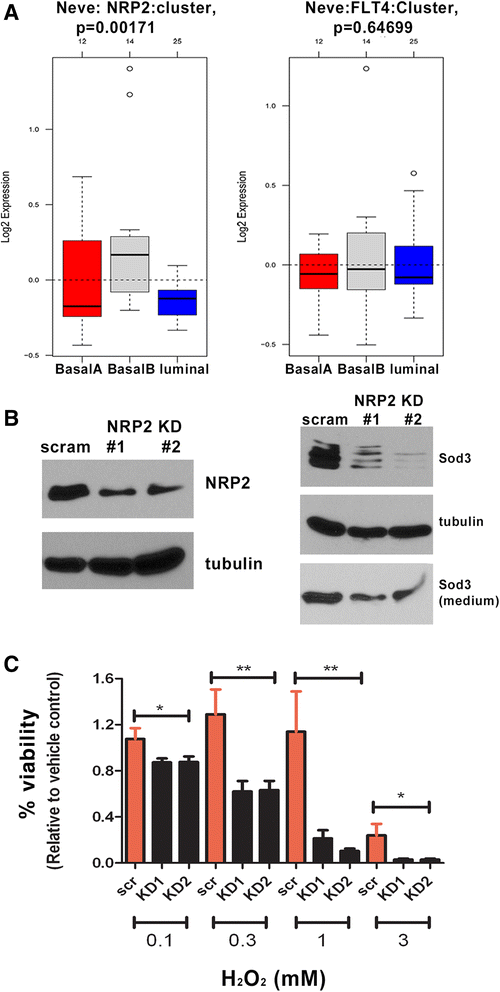

Supplement: Supplementary file 19 — Authors’ original file for figure 7 [file 13058_2014_462_MOESM19_ESM.gif]

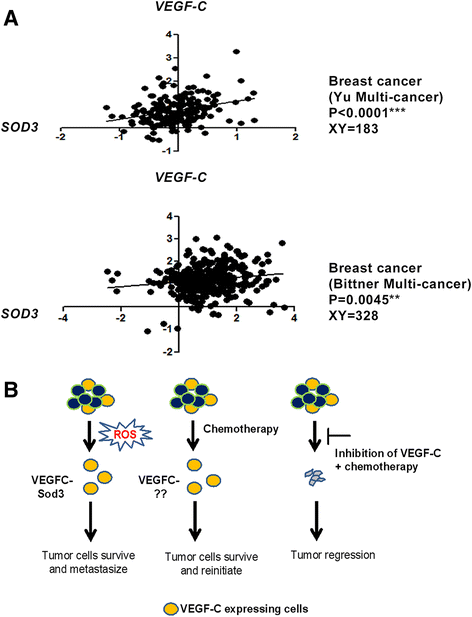

Supplement: Supplementary file 20 — Authors’ original file for figure 8 [file 13058_2014_462_MOESM20_ESM.gif]
